# Unveiling new interdependencies between significant DNA methylation sites, gene expression profiles and glioma patients survival

**DOI:** 10.1038/s41598-018-22829-1

**Published:** 2018-03-13

**Authors:** Michal J. Dabrowski, Michal Draminski, Klev Diamanti, Karolina Stepniak, Magdalena A. Mozolewska, Paweł Teisseyre, Jacek Koronacki, Jan Komorowski, Bozena Kaminska, Bartosz Wojtas

**Affiliations:** 1 Institute of Computer Science, Polish Academy of Sciences, Warsaw, Poland; 2 Department of Cell and Molecular Biology, Uppsala University, Uppsala, Sweden; 3 Nencki Institute of Experimental Biology, Warsaw, Poland

## Abstract

In order to find clinically useful prognostic markers for glioma patients’ survival, we employed Monte Carlo Feature Selection and Interdependencies Discovery (MCFS-ID) algorithm on DNA methylation (HumanMethylation450 platform) and RNA-seq datasets from The Cancer Genome Atlas (TCGA) for 88 patients observed until death. The input features were ranked according to their importance in predicting patients’ longer (400+ days) or shorter (≤400 days) survival without prior classification of the patients. Interestingly, out of the 65 most important features found, 63 are methylation sites, and only two mRNAs. Moreover, 61 out of the 63 methylation sites are among those detected by the 450 k array technology, while being absent in the HumanMethylation27. The most important methylation feature (cg15072976) overlaps with the RE1 Silencing Transcription Factor (REST) binding site, and was confirmed to intersect with the REST binding motif in human U87 glioma cells. Six additional methylation sites from the top 63 overlap with REST sites. We found that the methylation status of the cg15072976 site affects transcription factor binding in U87 cells in gel shift assay. The cg15072976 methylation status discriminates ≤400 and 400+ patients in an independent dataset from TCGA and shows positive association with survival time as evidenced by Kaplan-Meier plots.

## Introduction

There is a growing evidence that molecular markers such as *IDH1/2* mutations, *MGMT* promoter methylation, *TERT* promoter mutational status or 1p/19q co-deletion are important for diagnosis and prognosis of glioma patients^1^. However, there is an increasing need for more precise description of patient genetic background to better predict their survival and response to therapy. The majority of the recently discovered molecular patterns in human gliomas have been based on gene expression and methylation analysis of CpG sites^2,3^. Many of these patterns cluster into subtypes which allows a categorization of glioblastoma samples from The Cancer Genome Atlas (TCGA). Glioblastomas (GBMs), the most common malignant brain tumors in adults, have been divided into major subtypes: classical, mesenchymal and proneural^2^ based on transcriptomic analyses. These subtypes have been characterized by high frequency of specific somatic alterations, e.g. proneural tumors are enriched in *IDH1* mutations, while classical ones are enriched in *EGFR* amplification and *CDKN2A* deletions^2^.

Methylation status of specific genomic regions, such as promoters and enhancers, may activate or repress their activity^4^. Indeed, aberrant methylation of CpG islands in promoters of tumor suppressor genes in cancer is a phenomenon known for a long time^5^. CpG island methylator phenotype (CIMP) was first described in colorectal cancer^5^. More recently, methylation array platforms have been used to identify differentially methylated regions in other tumor types, including glioblastomas (G-CIMP)^6^. Glioma specific CpG island hypermethylation has been related to favorable survival prognosis and associated very closely to *IDH1/2* mutation in WHO grade II/III glioma and secondary glioblastomas^6,7^. Determinants of long-term survival of *IDH1/2* wild-type GBM patients beyond *MGMT* promoter methylation remain to be identified. Moreover, there is a subset of *IDH1/2* mutated G-CIMP phenotype GBM patients with a very poor prognosis^3^. Independent genome-wide DNA methylation profiling of short- (<1 y) and long-term survivors (>3 y) with the HumanMethylation450 K array has confirmed a G-CIMP positive phenotype that was tightly associated with the *IDH1* mutation and has identified a set of differentially hypermethylated CpG loci between long and short term GBM survivors, including members of the *HOX* genes, and *NR2F2* and *TFAP2A* genes coding for the transcription factors^8^. A recent study^9^ has found *LOC283731* promoter hypermethylation that has correlated with improved patient outcome. Its prognostic performance has been confirmed in three independent cohorts. LOC283731 promoter hypermethylation has been proposed as a prognostic biomarker in *IDH1* wild-type/non-G-CIMP GBMs^9^.

Though most of the previous analyses of DNA methylation patterns in gliomas have been performed on the TCGA datasets^10^, we aimed to search further these datasets for molecular factors (hereafter features) having impact on survival of glioma patients. Our analysis was built upon the use of the Monte Carlo Feature Selection and Interdependencies Discovery (MCFS-ID) algorithm that allows to perform supervised feature selection; for a brief account see Methods^11,12^. MCFS-ID identifies features and possible interdependencies between them that distinguish patients belonging to different classes, e.g. controls vs. sick.

Here, we aimed at discovering a set of significant features, such as gene expression profiles and DNA methylation sites, and their interdependencies that would enable accurate distinction between glioma patients with short and long overall survival (OS) i.e. days to death. Our study was based on the TCGA-derived data of 88 glioma patients diagnosed with WHO grades II, III, and IV, all of them with a full clinical information including time of death. Patients were assigned to one of the two decision classes depending on their OS: short-term survivors (less than 400 days: ≤400) and long-term survivors (more than 400 days: 400+). We did not take into account any “a priori” grouping of the patients, not even as WHO recommended classification by grades.

The discovered significant features are mainly differentially methylated DNA regions. Their significance was confirmed on an independent glioma study cohort. We confirmed that those features are much better predictors of patients’ OS than the previously described molecular markers (such as, e.g., *IDH, ATRX, DAXX* mutation status). Finally, we found that the most important methylation feature (cg15072976) overlaps with RE1 Silencing Transcription Factor (REST) binding site, is functional and its methylation status affects transcription factor binding in U87glioma cells as evidenced by gel shift assay.

## Results

### Feature significance and interdependencies

We applied our analysis pipeline to investigate putative associations in the dataset comprising both gene expression values and DNA methylation beta-values (β-values; Supplementary Table S1, see methods for details), as well as to obtain a ranking of significant features that accurately discern short and long overall survival of glioma patients, hereafter ≤400 and 400+ patients, respectively. The choice of such two decision classes was based on an analysis of the density of survival times ranging from 7 to 4084 days. The survival histogram can roughly be approximated by a power function with a negative exponent (Supplementary Fig. S1A), but a closer analysis of the histogram up to 1000 days revealed a consistent drop in its values for survival from about 400 days up (Supplementary Fig. S1B). This is in agreement with the results of the previous studies where the median glioblastoma survival has ranged from 10 to 16 months^13,14^.

The Monte Carlo Feature Selection and Interdependency Discovery (MCFS-ID) algorithm returns a ranked list of features that are the best, and thus play a significant role in the classification of objects that belong to different classes. It is capable of incorporating pairwise interdependencies, if there are such, between each of those best features and any of the other features. Moreover, within our approach no assumptions need to be imposed on the relationships between the features nor between the features and the classes the objects belong to. In particular, any nonlinear interdependencies are taken into account. Finally, the Interdependencies Discovery (ID) module is built into the algorithm. It returns a directed graph of the pairwise interdependencies found.

**Figure 1:**
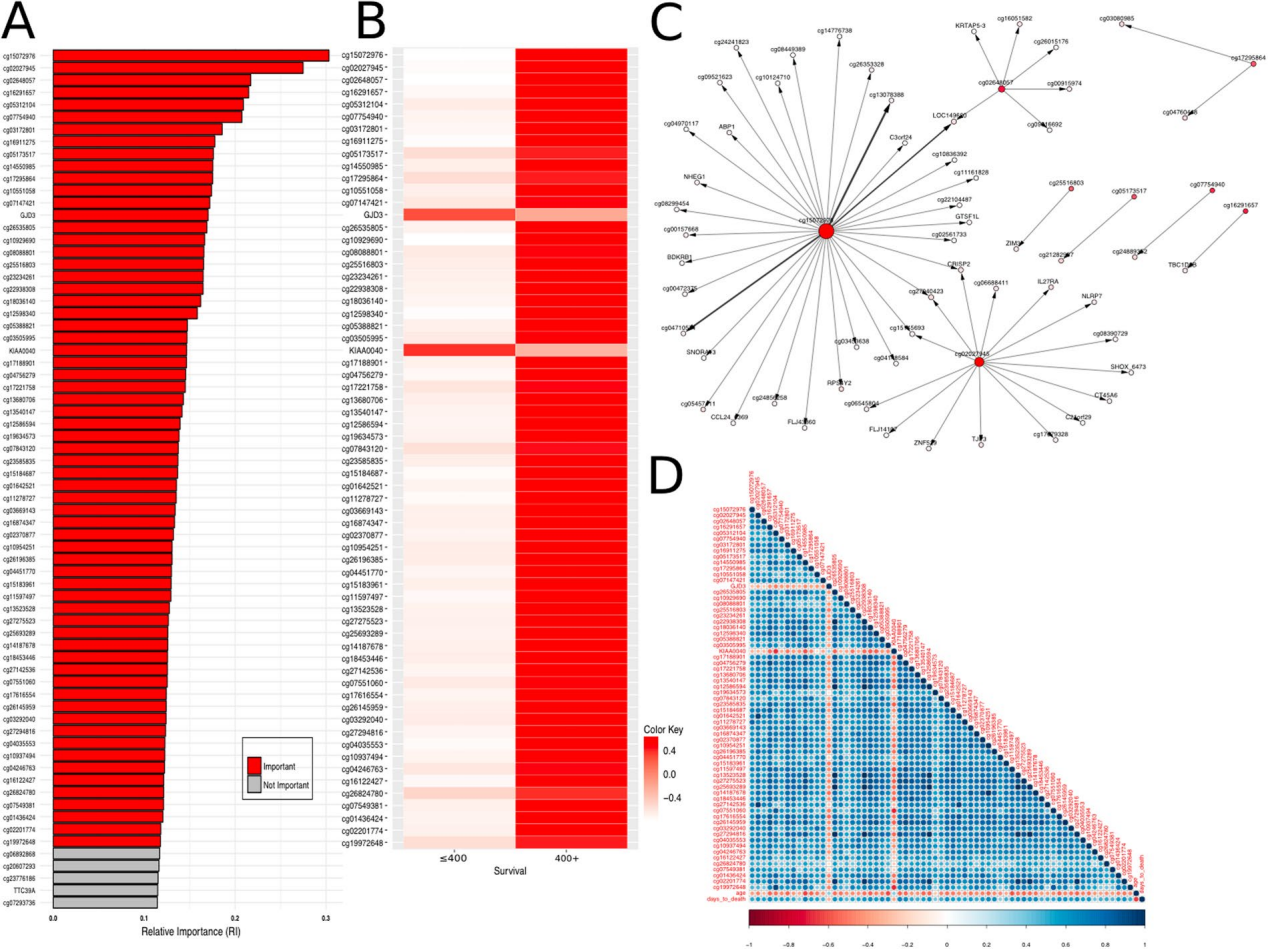
Significant features from the top of the ranking obtained by MCFS-ID. (**A**) Relative Importance (RI) of features placed in the top positions of the ranking. Red color corresponds to significant features, gray to those below the cutoff point (see Methods). (**B**) Mean values of the significant features for each of the decision classes (≤400 and 400+ patients). (**C**) Top 60 interdependencies detected by the MCFS-ID and visualized as a directed graph. The graph comes from structure analysis of the decision trees built by the MCFS-ID. Each node of the graph represents a feature which took part in constructing one or more trees (i.e., appeared in some splitting rules, each of which is set to include only one feature). When two features (more precisely, splits made on them) frequently co-occur along the (directed) paths of all decision trees, they are considered as interdependent and are connected in the graph by a directed edge with nodes representing these features. Herein, the higher the feature’s RI, the darker the red color of the corresponding node; the larger the size of a node the more edges related to this node; and, somewhat simplifying, the width and level of darkness of an edge joining two features are positively dependent on the number of the features co-occurrence and negatively dependent on the distances between the features along the paths they co-occur. (**D**) Pearson correlation matrix for MCFS-ID significant features, age and the outcome ‘days to death’ (DtD).

Interestingly, out of the top 65 significant features obtained from MCFS-ID, 63 were DNA methylation sites and only two genes (Fig. 1A). All significant DNA methylation sites refer to CpG type and none to CpH. Using this set of significant features, we were able to assign patients to correct decision classes (≤400, 400+) with balanced accuracy from 80% to 90% depending on the classifier we used (Supplementary Fig. S2). The values of significant features exhibit a clear pattern: ≤400 patients have lower CpG β-values but higher expressions of *GJD3* and *KIAA0040* genes than those of class 400+ (Fig. 1B). The observed differences were statistically significant (p-values by Kruskal-Wallis test with Bonferroni correction, Supplementary Fig. S3). Notably, the detected pattern shows that each of the features taken alone can be considered a rather reasonable class predictor, and hence no significant (i.e., instrumental in achieving high classification accuracy) interactions between features should be expected. Indeed, Fig. 1B shows that each of the features stands alone as a reasonably good class predictor and hence does not need any other interacting feature to predict the class.

Given the top 65 features, we calculated Mutual Information between each of them and the survival time. Mutual Information (MI) is a nonparametric measure of nonlinear dependencies between features under study and therefore much more general than the traditional correlation coefficient. It should be noticed, however, that the importance of each feature for classification, as assessed by MI, is measured separately for each feature and thus possible interactions between features cannot be taken into account. In Supplementary Table S2, the 65 features’ MIs and Relative Importance (RI) returned by MCFS-ID were compared. Interestingly, some features (marked in grey) were recognized as significant by MCFS-ID and as non-significant by MI. This seems to corroborate the claim that MCFS-ID is able to detect subtle dependencies and interdependencies between features. To confirm the significance of the features found in our analysis (i.e., using the MCFS-ID algorithm) we carried out an additional analysis using the Multiple Survival Screening (MSS) algorithm^15^. The latter algorithm allowed us to assess reproducibility and stability of the chosen subset of features. The obtained results confirmed the relevance of the features selected by MCFS-ID (described in the Supplement).

The ID part of the pipeline provided a number of interdependencies between features that are significant for classification and those that are not. In the graph (Fig. 1C), 60 strongest pairwise interactions are shown. Interestingly, only eight of the 65 significant features (cg15072976, cg02027945, cg02648057, cg16291657, cg05312104, cg07754940, cg03172801, cg16911275, cg19972648) strongly interact with some other features, and no strong interaction between any two significant features was found. In this way, the conjecture stated earlier has been confirmed. We also checked (details not shown) that incorporation of features that strongly interact with the two significant features into the classification only marginally, if at all, improves classification accuracy.

Note that the above refers to predictive interdependencies between features, since the dependencies in question require the context of the two decision classes (≤400, 400+). Thus, a separate scrutiny of associations between the 65 significant features was needed. In Fig. 1D Pearson’s correlation matrix is given for 67 features: significant DNA methylation sites, two genes, patient survival (defined as days_to_death, hereafter DtD) and age. The two genes and age are negatively correlated with DNA methylation sites and DtD, whereas correlation between DNA methylation sites is positive. One may expect that stronger correlation is the result of a closer genetic distance. Hence, we focused next on the two chromosomes where the detected significant genes (*KIAA0040, GJD3)* are placed. On chromosome 1, there are 5 DNA methylation sites (cg16911275, cg12598340, cg04246763, cg16122427, cg01436424) along with the *KIAA0040* gene and two (cg11278727, cg10937494) on the chromosome 17 with the *GJD3* gene. No significantly stronger correlation between genes and DNA methylation sites from the same chromosome was observed for either *KIAA0040* or *GJD3*.

Furthermore, in order to better elucidate the significance of the identified DNA methylation sites, we assigned them to genes using level 3 TCGA 450 k Illumina Bead Array annotations. We found that 44 out of the 63 significant DNA methylation sites are paired with a corresponding gene, while the remaining 19 are not assigned to any gene. Interestingly, out of the 44 methylations, there are 7 sites in a range of 865 bp annotated to *MYADM* gene and two DNA methylation sites at a distance of 287 bp to each other are annotated to *TBR1*. The remaining 35 DNA methylation sites are assigned to 35 various genes. Moreover, we examined correlation between each of the DNA methylation’ β-values and an expression level of the corresponding gene. We found that the 12 methylation sites relatively strongly correlate with expression levels of the corresponding genes (Spearman correlation abs(rho) >0.5). All correlations are inverse, the strongest being that between cg14550985 and *RIN1*, and equal to (−0.78). In the remaining 11 pairs, only 8 genes appear, since five of the methylation sites are correlated with *MYADM* (Supplementary Fig. S4).

DNA methylation status may vary due to multiple factors, among them age and gender. Accordingly, we employed Interaction Information to verify whether age or gender affect dependence between the top 65 features and survival (see Supplementary Information for explanation). No significant interaction between age or gender and any of the top 65 features was found. Therefore, we may conclude that age and gender do not affect the relationship between the top 65 features and the survival (Supplementary Table S2).

### The relationship of newly discovered significant features to known molecular markers and clinical characteristics of patients

In order to better assess utility of the discovered significant features (N = 65), the MCFS-ID analysis pipeline was run again on data from the same 88 patients but now comprising known molecular markers as well as clinical characteristics (hereafter patient characteristics) adapted from Ceccarelli *et al*. (see Supplementary Information) and only the top 5 k features from the MCFS-ID ranking (Fig. 1A). Among the set of characteristics there were several predictors of patient’s survival, e.g. *IDH, ATRX* and *DAXX* mutation status, methylation of *MGMT* promoter and *TERT* promoter status*, TERT* expression. In the set of patient characteristics, a WHO tumor grade was also included. As expected patient’s survival was different in patients with tumors of different grades (Supplementary Fig. S5). However, molecular markers were recognized as more significant features for predicting patient survival than the tumor grade.

All patients’ characteristics as well as the top 5 k features were used to verify their significance for distinguishing the patients with different OS (≤400 or 400+). It turned out that the highest position taken by any of the patients’ characteristics in the new ranking is 137 and belongs to ‘IDH.codel.subtype’. The overlap between the top 65 features from the first ranking (Fig. 1A) and the one from the second MCFS-ID analysis (Supplementary Fig. S6) was equal to almost 74% (Supplementary Fig. S7). Moreover, the two sets of the top 25 features coincided, thus the reliability of the procedure was confirmed. In summary, it showed that– at least for the presented data – the features discovered by our pipeline (63 methylation sites and two genes) are much better predictors of patients’ outcome, than any of those earlier reported in the literature.

### Genomic Annotations of significant DNA methylation sites

**Figure 2:**
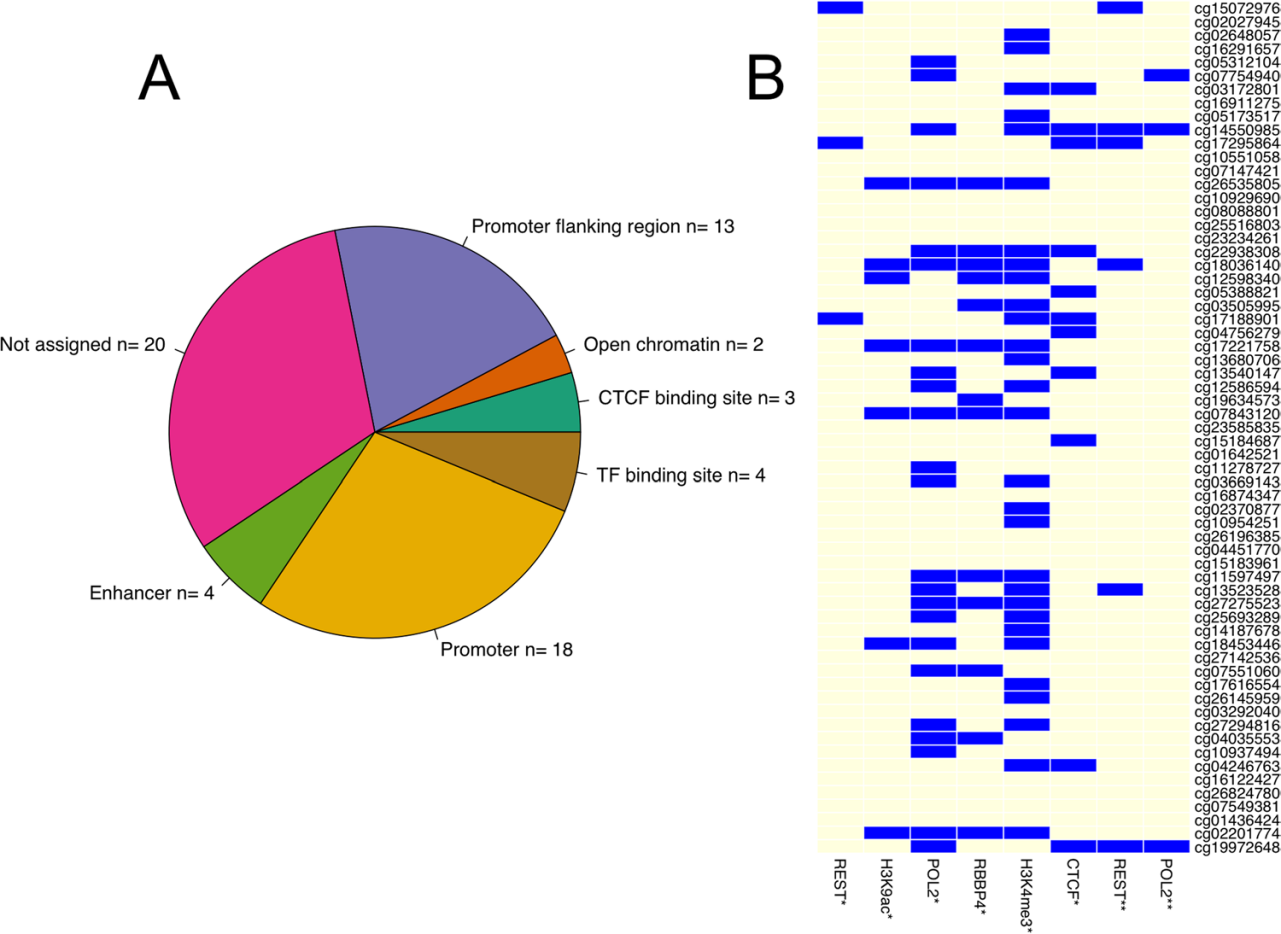
Distribution of Methylated Regions (MRs) annotated to specific genomic regions. (**A**) Annotated with the use of biomaRT tool or (**B**) with the use of data deposited in NCBI (marked with*; for aliases see Supplementary Table S4), as well as ENCODE (marked with**).

After ensuring that the selected 65 features are capable of predicting the decision class of the patients with high accuracy, we aimed at verifying their participation in molecular processes. For that reason, we determined the annotations of the genomic regions surrounding the 63 methylation sites. We extended each methylation site by 25 bp upstream and downstream, constructing Methylated Regions (MRs) 51 bp long. Using biomaRT^16^ we found that most of the MRs occurred in promoters or promoter flanking regions. Interestingly, almost one third of the MRs were not associated to any specific region (Fig. 2A). All MRs were assigned to a single element except cg17295864 that was marked with both CCCTC-binding factor (CTCF) and enhancer (11th in the MCFS-ID ranking). The MRs that intersected with brain-specific, neuron-specific, neuronal stem cell specific and astrocyte-specific enhancers obtained from FANTOM, returned only the cg03505995 methylation site, which was annotated to a CTCF region by Ensembl^17^. We also identified MRs intersecting with various ChIP-seq signals in five glioma-related cell lines in ENCODE and NCBI (Fig. 2B). These signals include histone modifications H3K4me3 and H3K9ac that are associated with active gene promoters, transcription initiation and elongation. Additionally, the genomic signals of CTCF, REST, the RB binding protein 4 (RBBP4) and POL2 (Fig. 2B) were overlapping our top MRs. The REST binding sites that overlapped MRs come from U87 human glioma cells. Methylation sites from those MRs were ranked as 1^st^, 10^th^, 11^th^, 21^st^, 26^th^,46^th^, and 65^th^ by the MCFS-ID. According to Ensembl, no annotation could be assigned to three of those methylations (1^st^, 10^th^, 26^th^). Nevertheless, the first one occurred 822 bp upstream from the coding region of *GAL3ST2*, the second one overlapped with the *RIN1* gene region and the last one with *KCNH2* intron. The other REST intersecting methylation sites matched the following regions: CTCF binding site and enhancer, promoter flanking region, promoter and enhancer, respectively. It is worth mentioning that U87 POLII signals from ENCODE^18^ intersected with three MRs: the two already reported in the case of REST (10th and 65th) and a new one cg07754940 (6th in the ranking). Based on NCBI data one can find much more POL2-methylation intersecting regions. The intersection results of functionally active genomic regions of glioma-related cell lines, especially U87 with MRs, as well as the important genomic functions annotated to them, suggested that the methylation status of those cytosines may play a significant role not only in classification but also in a wider spectrum of molecular interactions.

### Local epigenomic landscape of the top DNA methylation site

**Figure 3:**
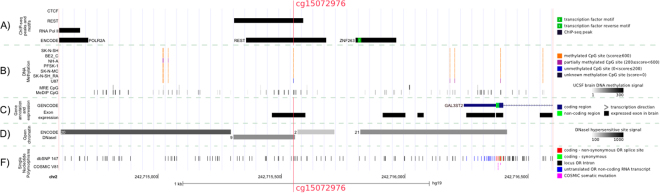
Epigenetic landscape associated with the most indicative methylation site cg15072976 in U87 glioma cells. (**A**) The first three tracks show ChIP-seq transcription factor (TF) binding sites (TFBS) curated from brain cell lines for CTCF, REST and RNA Pol II (cf. Methods). The fourth track shows ChIP-seq TFBS from ENCODE including Factorbook motifs. This track is a curated set of various transcription factors (161) from a collection of tissues and cell lines. (**B**) The first seven tracks shown the methylation level of the CpG sites in the cg15072976 region for seven human brain cell lines measured by 450 K bead Illumina methylation array from ENCODE/HAIB. All cell lines (SK-N-SH, BE2 C, PFSK-1, SK-N-MC, SK-N-SH RA and U87) are cancerous, except for normal human astrocytes NH-A). As score is used the β-value of the methylation multiplied by 1000. The last two tracks show the methylation level of healthy brain tissue from Methylation-sensitive Restriction Enzyme digestion followed by sequencing (MRE-seq) and MeDIP-seq in grayscale^34^. (**C**) The first track displays genes from GENCODEv19^35^. The second track shows the microarray exon expression from 13 regions of the human late mid-fetal brain. (**D**) Open chromatin landscape. A normalized score (0–1000) was computed for all the peaks of DNaseI hypersensitive sites for 125 cell lines (1% FDR). The peaks were clustered by score and clusters with score less than 100 were removed. The extend of the box indicates the length of the cluster, the color is a grayscale proportional to the highest signal observed in any cell line and the number left from each box indicates the number of contributing cell lines. (**E**) The first track shows SNPs and indels from dbSNP build-147^36^. The second track contains SNPs from somatic non-inherited mutations curated from a large number of sources from the Catalogue of Somatic Mutations in Cancer (COSMIC) version81^37^.

We investigated the genomic landscape of the topmost feature from the MCFS analysis. First, we observed that cg15072976 overlaps with the binding site of the transcription factor (TF) REST which is predicted by the analysis of the curated TF binding sites (TFBS) and the ENCODE predicted motifs (Fig. 3A). The high methylation level of the CpG site was confirmed in a collection of six brain related cell lines and by Methylation Dependent Immunoprecipitation followed by sequencing (MeDIP-seq) (Fig. 3B). Only the U87 glioma cells show an unmethylated status for cg15072976. This CpG site also overlapped with open chromatin sites and intergenic single nucleotide polymorphisms (SNPs) indicating its potential functionality (Fig. 3D–E). Additionally, we found this site in the promoter of *GAL3ST2*, which encodes a member of the galactose-3-O-sulfotransferase protein family (Fig. 3C). *GAL3ST2* has been known to be expressed in several brain tissues (GTEx average FPKM score for brain tissues 150 – data not shown) and its downregulation has been associated to human colonic non-mucinous adenocarcinoma^19^. However, we did not observe any significant difference in the *GAL3ST2* expression between the groups ≤400 and 400+ (Supplementary Fig. S8). We focused on elucidating how cg15072976 methylation status affects the REST binding site.

### Confirmation of functional significance of the cg15072976 methylation site

**Figure 4:**
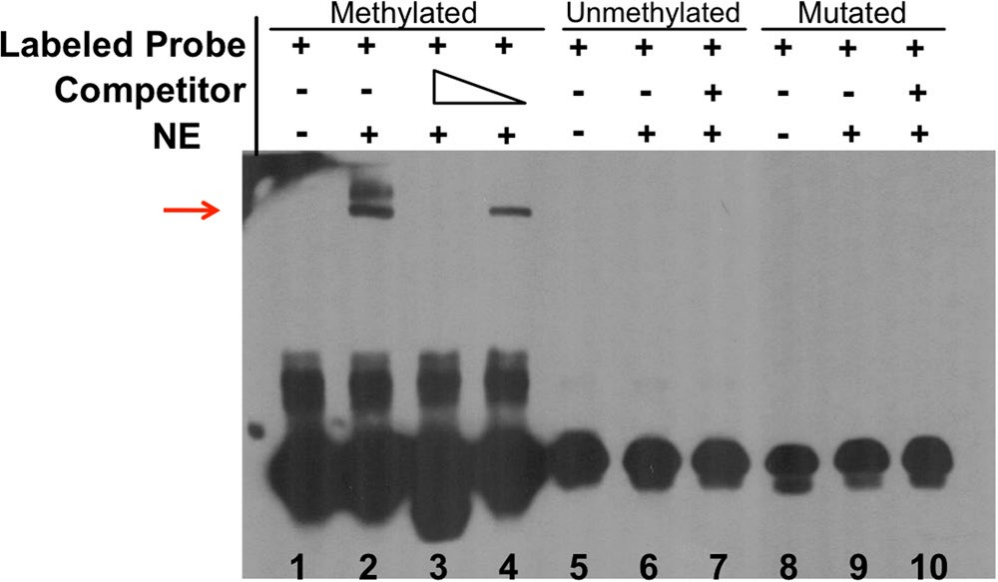
Binding of nuclear proteins from U87 glioma cells to DNA sequence containing REST consensus motif with methylated CpG site detected with electrophoretic mobility shift assay. Three variants of biotin-labeled, double stranded DNA probe carrying the REST consensus motif were used in the experiment: methylated or unmethylated CpG site or a probe carrying CG → AT nucleotide substitution. Competition assays with the excess of the corresponding, unlabeled probe were performed (competitor). For the methylated probe 2 100× or 20× molar excess of the competitor was added to confirm specificity of the binding. The control lanes with no nuclear extract (NE) added (lanes 1, 5 and 8) show migration of a free probe. The reaction mixtures were resolved in 4% native electrophoresis gels.

From the six MRs overlapping with U87 REST peaks we selected the one that represented the top-most feature from the MCFS-ID ranking. Using FIMO^20^ we detected transcription factor motifs for REST overlapping with the MR. Thus, we obtained not only information about overlapping REST binding site from ENCODE U87 data, but also confirmation of the existing REST binding motif. To test the functionality of methylation status for this site we performed Electrophoretic Mobility Shift Assay (EMSA) (Fig. 4). Biotin-labeled DNA probes containing methylated or unmethylated CpG site were incubated with nuclear extracts isolated from U87 glioma cells; the binding in the absence or presence of an excess of an unlabeled analogue (competitor) served as a specificity control. DNA-protein complexes were formed exclusively for methylated consensus sequence manifested by retarded gel mobility. The 200-fold molar excess of unlabeled probe had out-competed specific interactions and 20-fold molar excess of competitor reduced but did not fully eliminate a positive shift. A probe containing CG → AT nucleotide substitution in the methylation site was used as an additional negative control.

Each patient-derived cell line displays different molecular background. Two commonly used in *in vitro* studies glioma cell lines, U87 and LN18, exhibit some molecular differences such as opposite *MGMT* promoter methylation status as well as *TP53* and *PTEN* mutation status^21^. Due to this fact we investigated whether the top methylation site occurring at the consensus REST motif is important for its binding in an additional glioma cell line (LN18). EMSA was carried out identically as above, but this time with the LN18 nuclear extract (Supplementary Fig. S9). As expected, EMSA results showed the same pattern as in U87 cells indicating that the binding of REST to the methylated site cg15072976 is commonly affected in malignant gliomas.

### Prediction of REST-DNA complex structure

**Figure 5:**
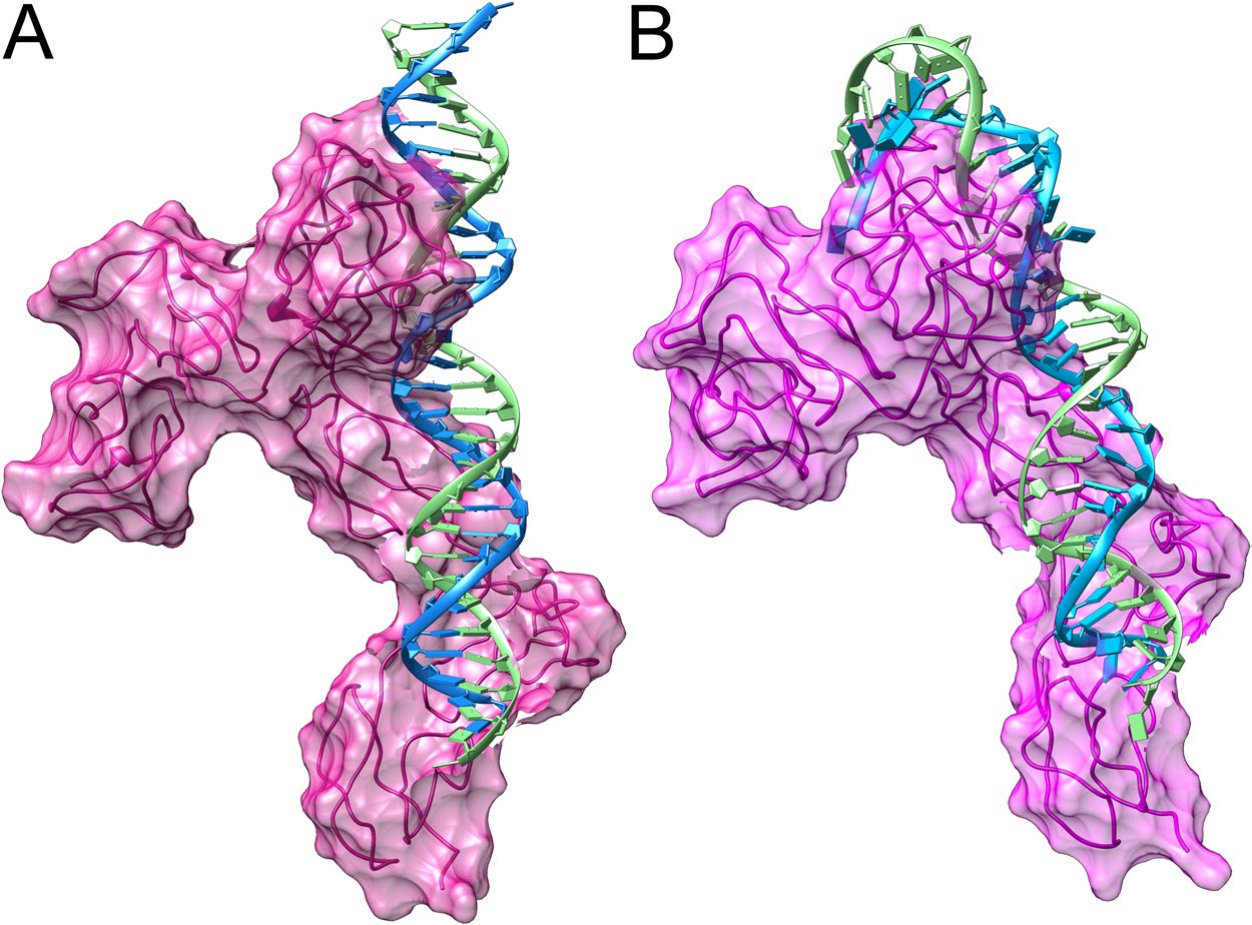
Computational prediction of specific DNA sequence containing the REST motif and the REST N-terminus fragment. DNA-binding motif docked to the REST with calculated surface of the REST. (**A**) Rigid model after energy-minimization. (**B**) Dynamic after short molecular dynamic simulation.

The structure of the REST protein has not been solved experimentally yet. Therefore, we employed structure bioinformatics approach to protein structure prediction^22,23^. We found REST to be moderately similar to other DNA-binding proteins. In particular, its N-terminal fragment that includes amino-acids residues from ~150 to ~430 was highly similar to its counterparts in other proteins. Consequently, the structure of the REST part that interacts with the specific DNA sequence (the same as used in the EMSA experiment) was predicted with relatively high reliability (see Supplementary Information for details). Using a template-based model, we predicted the rigid structure of the protein-DNA complex of a short specific DNA sequence containing REST binding motif and the REST N-terminus fragment (Fig. 5A). Upon molecular dynamic (MD) analysis, relaxation of the complex was observed. Major structural changes of both DNA and REST N-terminal caused by their strong interactions were found (Fig. 5B). The DNA binds to the zing-finger regions of the REST and during interaction its structure was subjected to bending from the perfect B-DNA conformation, while protein moved to grab the DNA more tightly, especially in the major groove regions of the DNA. We concluded that: (i) the selected, specific DNA sequence with REST motif bound strongly to the REST N-terminal part confirming our hypothesis; (ii) the structure obtained was a reliable starting structure that occurred in the binding process; (iii) full-blown MD studies as well as a thorough analysis of methylation influence on the complex formation are needed to show the likely structure of the complex and reveal the mechanism of binding and the specificity of the DNA-protein binding site.

### Validation of feature selection results on an independent dataset

Our MCFS-ID analysis was performed using the TCGA dataset collected in 2015, 88 patients, from now on termed the training set. Currently, the TCGA dataset with matching RNAseq, 450 k methylation arrays and OS time, comprises additional 79 new patients, from now on termed the test set. Inclusion criteria for both training and test set was a confirmed status of patient being deceased.

**Figure 6:**
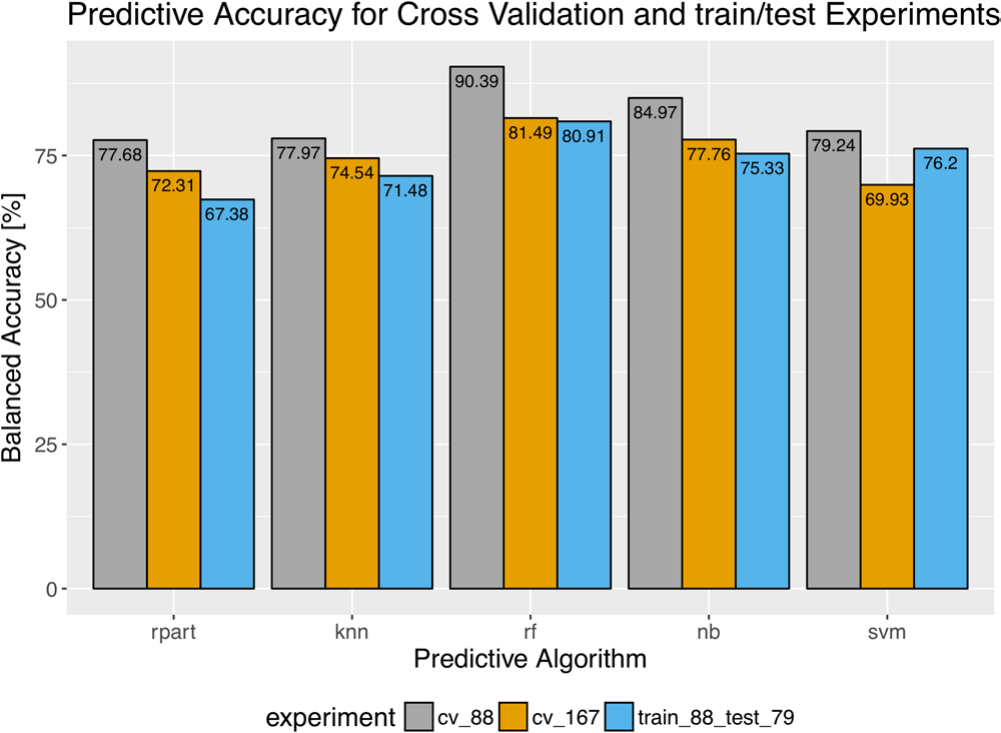
The balanced accuracy values that result from the three Cross-Validation (CV) experiments: gray and orange corresponds to 10-folds CV on the original 88 and all 167 patients, respectively; blue corresponds to the most reliable result, where the set of 88 patients was used for training and the remaining 79 patients for testing; rpart-decision tree, knn- k Nearest Neighbours, rf - random forest, nb - naive Bayes, svm - support vector machines.

In order to validate our 65 top-ranking features we performed 3 different experiments. In the first experiment a 10-fold Cross-Validation (CV) was used on the 88 patients. Secondly, 10-fold CV was performed on the whole data comprising 167 patients. Thirdly, we used the training set (n = 88), for model building, and tested the model on the test set (n = 79). The obtained CV balanced accuracy results are presented in Fig. 6. It is not surprising that the highest balanced accuracy is obtained for the set of 88 patients because it is the original data used for feature selection. However, the most reliable experiment (testing on the unseen 79 patients) also led to a successful prediction with a high balanced accuracy equal to 80.91% for the random forest classifier (Fig. 6).

**Figure 7:**
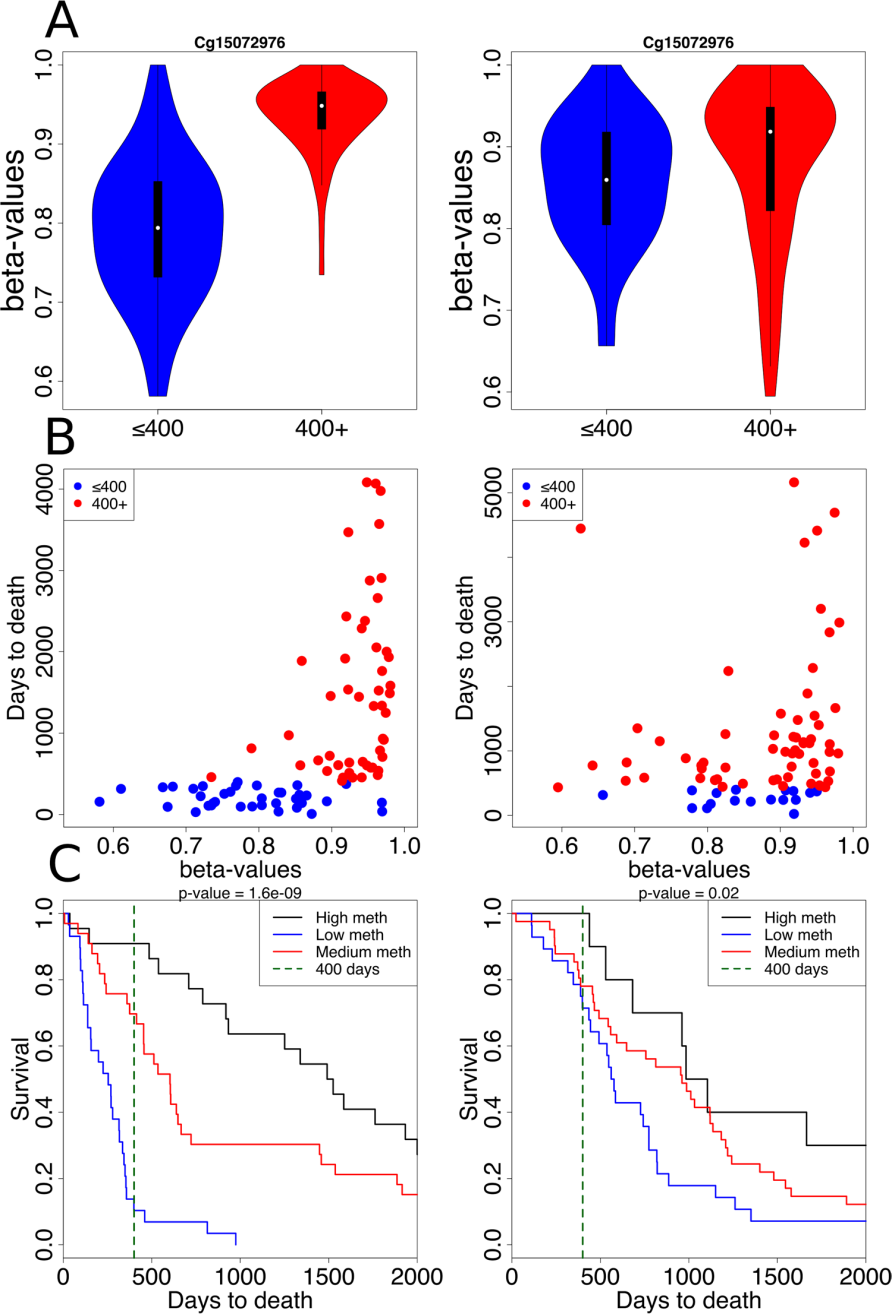
Validation results of cg15072976 performance on both training and test sets. Upper panel: Distributions of cg15072976 β-values presented as violin plots for ≤400 (blue) and 400+ (red) patients from the training set (**A**) and test set (**B**). Middle panel: dot plots of cg15072976 β-values for the training set (**C**) and test set (**D**). Lower panel: Kaplan-Meier plots for patients from the training set (**E**) and test set (**F**), with patients divided according to their cg15072976 β-values; black line marks 400 days, chi square p-values are written on the top of each panel.

We have also used the test set to validate performance of our top cg15072976 methylation. The difference between the methylation level distributions for the two classes of patients (≤400 and 400+) was most obvious in the case of training data (Fig. 7A). This difference, though not so significant, was maintained for the test data (Fig. 7B). Positive association between methylation level of the cg15072976 site and survival time for both training and test sets could also be readily inferred from corresponding dot and Kaplan-Meier plots (Fig. 7C–F, respectively).

## Discussion

The majority of the samples from TCGA glioma datasets (GBMs, LGGs) have been processed using Illumina’s Infinium Human Methylation k27 BeadChip that covers over 27,000 CpG methylation sites. A part of the glioma samples from TCGA have been processed with Illumina’s Infinium Human Methylation k450 BeadChip that, as described by the vendor, covers 96% of known CpG islands. Apart from covering most of the methylation sites within the known CpG islands, it also covers: (1) CpG sites outside of CpG islands, (2) non-CpG methylated sites identified in human stem cells, (3) differentially methylated regions (DMRs) identified in human cancer-normal tissue pairs, (4) CpG sites outside of coding regions, (5) miRNA promoter regions. CpG sites within CpG islands have been quite well described and their functional importance for a proximal gene expression is well understood. Interpretation of functional importance of DMRs located far away from the genes is difficult. It is even more difficult to assess the importance of methylated cytosine sites that are followed by a base different from G (CpH)^24^. The last one was not our case because all 63 significant DNA methylation sites were of the CpG type. Having access to ChIP-seq data from glioma cell lines, we demonstrated that the significant methylations could be involved in transcription regulation. Methylation at a specific site may inhibit protein binding to DNA similarly to SNP appearing within TFBS, as shown for CTCF^25^. At the same time, the methyl-CpG-binding domains (MBD) of various proteins, e.g., MeCP2^26^ bind with a high affinity to MRs protecting DNA from other TFs. Furthermore, MBD-containing proteins may recruit other molecules, e.g., histone deacetylases, chromatin remodeling factors that change chromatin accessibility for TFs. Interestingly, in our study only in a case of some genes their expression corresponded with the level of CpG methylation (Supplementary Fig. S4). This supports a hypothesis about a relatively small effect of a single regulatory region if a gene is regulated by combinatory action of several of them. In such a case, regulatory regions should be considered jointly to detect their association with gene expression levels^27^. Such approaches are beyond the scope of this study, since it requires more genomic and epigenomic data as well as a larger patient’s cohort than available.

Nevertheless, it has been recently confirmed that methylation status of tumor cells is crucial for patients’ survival. Firstly, the G-CIMP phenotype has been described and promoter methylation of oncogenes has been confirmed as a good prognostic factor^6^. Moreover, G-CIMP methylation status adds a prognostic value to the existing prognostic markers, such as *IDH1/2* mutations, 1p-19q codeletion and *MGMT* promoter methylation^3^. It has been shown that a subgroup of *IDH1/2* positive gliomas with low G-CIMP profile has a shorter overall survival than other *IDH1/2* positive gliomas^3^. While it is quite obvious that G-CIMP methylation status does have a clinical meaning, it is hard to apply G-CIMP methylation evaluation in a clinical setting. There is a need to specify a limited set of methylation markers that can be successfully introduced into clinic.

TCGA methylation datasets comprise, as it was described before, of both 27 k and 450 k methylation arrays. In a work of Ceccarelli *et al*. a common set of methylations between 27 k and 450 k was used to assure a reliable data size. As 27 k methylation array contains only CpG methylation sites, mostly from promoter regions, these methylation sites have a relatively easy interpretation, since hypo-/hypermethylation of promoter sites is a well known mechanism of gene expression regulation. In our work, we undertook a more demanding path and considered only 450 k methylation arrays from glioma samples deposited in TCGA that had a matching transcriptomic profile from RNAseq and clinical records (patients’ OS and patients’ status as ‘deceased’ was the inclusion criterion). We wanted to confirm MCFS-ID utility in analyzing large datasets (with approximately 0.5 M features) and attempted to discover biologically valid findings. Interestingly, out of 63 top DNA methylation sites in our ranking only 2 were present on both 27 k and 450 k methylation arrays, which confirmed that selecting a larger dataset was reasonable. We described their putative role in molecular processes by assignment to specific genomic regions, genes, and described local molecular landscape. Among the most interesting findings, we reported the *MYADM* gene related to multiple methylated sites as well as *TBR1*. This also applies to the *RIN1* gene whose expression had the highest correlation with β-value of the CpG (the 10^th^ position in the ranking) and overlapped with both REST and POLII of U87 glioma cells.

Summarizing, we demonstrated a proof of principle that MCFS-ID was able to find a number of significant features (Fig. 1) that with a high accuracy predicted patient’s survival. It is worth to notice that all significant features discovered in our pipeline were better predictors than the previously reported ones, e.g. *IDH1/2* status. Moreover, these significant features were mapped to functionally active genomic regions (Figs 2 and 3) and the biological function of the most top one was confirmed with a biochemical gel shift assay (Fig. 4). Finally, our top-most DNA methylation site - cg15072976 - was validated in an independent set of 79 samples from TCGA, and was found to predict accurately patients with better prognosis (Fig. 5). Importantly, first and second top DNA methylation sites had relatively high and narrow distribution of the β-values. It could be a reason why commonly used discretization methods^28^ would overlook these putative prognostic markers. All values would be assigned as “high” losing the inner variability. We have to keep in mind that tumor is a mixture of different cells with different DNA methylation and expression patterns. The experimental results reflect their cumulative effect.

In a long-term perspective, we would like to test the utility of selected DNA methylation sites as markers of response to treatment. A limiting factor in our analysis is that patients from TCGA have been treated in a number of clinical trials with different combinations of drugs and this affects patients OS. Despite of that, we were able to find relevant prognostic DNA methylation site that may affect REST transcription repressor binding to DNA (Figs 4 and 5). It would be desirable to discover methylation-based signatures that predict patient’s survival or recurrence as had been done e.g. for colorectal cancer using gene-expression signatures^29^. Unfortunately, in the case of significant methylations that we have found there is not enough data to detect valid signatures. Hopefully, new large next generation sequencing projects will supply data needed to reach this aim

### Final conclusions

In conclusion, we demonstrated the effectiveness of MCFS-ID approach in finding biologically/clinically relevant features, such as cg15072976 DNA methylation site, which was proved to be a good predictor of patient’s survival. It was experimentally evidenced that methylation of this site most likely affects binding of the REST transcription factor to DNA. As our most important features were found in the 450 k methylation dataset, but not within the k27 methylation dataset, we propose that larger datasets containing non-classical CpG methylation sites may reveal important clinical features and we should be careful not to overlook them by simplifying analyses.

## Methods

All methods were carried out in accordance with relevant guidelines and regulations.

### (MCFS-ID)

Given a set of objects, each of which is described by a vector of features and is known to belong to a particular class out of an *a priori* determined set of classes, the main task is in building a classifier capable of properly assigning yet unseen objects into proper classes. Monte Carlo Feature Selection and Interdependencies Discovery (MCFS-ID) algorithm is a novel method for ranking features from high dimensional data according to their importance for a given classification task, regardless of a classifier to be later used, as well as for discovery of linear and nonlinear feature interdependencies. This goal is achieved through constructing thousands of decision trees (Supplementary Fig. S10). The trees are constructed on randomly selected subsets of features and objects. A particular feature is considered to be important, if it is likely to play a significant role in the process of classifying objects into classes “more often than not”. This “readiness” of a feature to take a part in the classification process, termed relative importance (RI) of a feature, is measured via structure analysis of the constructed decision trees^11^. If the data contains a set of features that can be used for successful classification, the algorithm returns them at the top of the ranking after having performed a number of iterations needed for the algorithm’s convergence. Since the ranking as such does not enable one to discern between important or informative and not important features, a cutoff between these two types of features has been proposed^12^.

The structure analysis described above enables making the algorithm return a directed graph of feature interdependencies^12^. In short the algorithm identifies features that “cooperate” in determining that some objects belong to one class, another objects to another class, and so on. Thus, our way to discovery of feature interdependencies rests on determining multidimensional dependence between the classes and sequences of features (as stated in the Introduction, the interdependencies sought can be termed contextual or predictive).

### Data analysis

Our analysis pipeline included the Illumina’s Infinium Human Methylation k450 BeadChip data as well as RNA-seq and clinical records provided by the TCGA for 88 glioma patients (tumors were diagnosed as WHO grades II, III gliomas and grade IV glioblastomas) (Supplementary Table S1). Data from TCGA were uploaded as normalized Level 3 data for both RNAseq and methylation data, FPKM values were used for RNAseq, and β-values for methylation; no additional data processing regarding technical batch correction was applied. After elimination of zero variance features, our decision system consisted of gene expression levels for 19943 genes and pseudogenes, β-values of 396065 DNA methylation sites and clinical records including tumor grade, gender, and age of a patient. Additionally, binary decision for each patient was added depending on his or her days to death information (Supplementary Fig. S1). They were grouped as those who survived up to 400 days (n = 38) hereafter ≤400 patients and those who survived at least 401 days (n = 50) hereafter 400+ patients. The achievement of project objectives required a reduction of the original data complexity without loss of informative features. To this end we performed feature selection using the MSFS-ID method implemented in the *rmcfs* package: (https://cran.r-project.org/web/packages/rmcfs/index.html). When running *rmcfs* we chose the following parameter settings: number of feature subsets (*s*) equal to 50,000; number of features (*m*) in each subset equal to 500; number of decision trees (*t*) built for each subset equal to 5. The remaining parameters remained set to their default values.

### Experimental verification of TF binding sites and Electrophoretic Mobility Shift Assay (EMSA)

To assess whether the top feature cg15072976 methylation might have functional implications for binding a protein to DNA, we examined transcription factors (TFs) that were reported to bind to DNA at this position. From the Encyclopedia of DNA Elements (ENCODE) we learnt that in the U87 astrocytoma cell line there has been reported a REST binding site overlapping with our top feature DNA methylation. Using Find Individual Motif Occurrences (FIMO) v. 4.11.2^20^ we confirmed that there are possible REST motifs overlapping with cg15072976. In view of these results the oligonucleotide sequences were designed for the purpose of further molecular analysis using Electrophoretic Mobility Shift Assay (EMSA).

Nuclear extracts from U87 and LN18 human cells were prepared as previously described^30^. The Bradford method was used to determine protein concentration. DNA probes containing REST consensus motif with methylated, unmethylated or mutated (CG to AT nucleotide substitution) CpG site were generated by annealing sense and antisense oligonucleotide (synthesized by Genomed, Poland) in the order as presented in the Supplementary Table S3 (95 °C to 25 °C room step-down).

Nuclear extracts (2 µg of nuclear proteins) and 200 fmol of biotin labeled probe were incubated in a binding buffer (10 mM TRIS pH 7.5, 50 mM KCl, 1 mM DTT, 2.5% glycerol, 5 mM MgCl2, 1 µg Poly (dI·dC), 0.05% NP-40) for 20 min at room temperature. For the competition assays, 40 pmol of unlabeled probes were added to the binding reaction (or 2 pmol, if applicable). Reaction was stopped by adding a gel loading buffer, then samples were electrophoresed in non-denaturing 6% polyacrylamide mini gel (8 × 8 × 0.1 cm) in TBE buffer and electro-transferred to nylon membrane (Thermo Scientific, cat. no 77016). Complexes of DNA and proteins were cross-linked to the membrane using UV-light crosslinking instrument (Ultra Lum) and detected by chemiluminescence using LightShift Chemiluminescent EMSA Kit (Thermo Scientific).

### Peak-calling and curation of glioma ChIP-seq experiments

Here, our starting point was a thorough review of literature on ChIP-seq experiments performed in glioma-related cell lines. The goal was to accumulate all the TF binding sites and histone modification signals that co-occurred with methylation sites discriminating between ≤400 and 400+ glioma patients. We curated 40 ChIP-seq experiments for 5 different cell lines (Supplementary Table S4). For the quality control and the peak calling we used the ENCODE3 pipeline^31^ as implemented by Kundaje Lab. Due to the lack of at least two replicates per experiment and/or controls we did not use the irreproducible detection rate (IDR) option, but the simple overlap. For the curation of ChIP-seq experiments from different cell lines we implemented a simple voting algorithm^32^.

### Kaplan-Meier analysis

Patients from both training and test sets were divided according to their cg15072976 methylation status to 3 classes: High meth (β-value > 0.96), Low meth (β-value < 0.85), Medium meth (0.85 ≤ β-value ≥ 0.96). Difference between survival curves was calculated by *survdiff* function from survival R package^33^. P-value was calculated between High meth and Low meth groups by log-rank test from the *survdiff* function.

## Electronic supplementary material

Supplementary information
