# Supplementary material for: Unveiling new interdependencies between significant DNA methylation sites, gene expression profiles and glioma patients survival
Source: Sci Rep. 2018 Mar 13;8:4390. doi: 10.1038/s41598-018-22829-1 (PMC5849697; doi:10.1038/s41598-018-22829-1)
Supplement: Supplementary file 1 — Supplementary information [file 41598_2018_22829_MOESM1_ESM.pdf]

Unveiling new interdependencies between significant DNA methylation sites, gene expression profiles and glioma patients survival

Michal J. Dabrowski[1*], Michal Draminski[1], Klev Diamanti[3], Karolina Stepniak[2], Magdalena A. Mozolewska[1], Paweł Teisseyre[1], Jacek Koronacki[1], Jan Komorowski[1,3], Bozena Kaminska[2] and Bartosz Wojtas[2*]

[1] Institute of Computer Science, Polish Academy of Sciences, Poland

[2] Nencki Institute of Experimental Biology, Warsaw, Poland

[3] Department of Cell and Molecular Biology, Uppsala University, Sweden

*corresponding authors: michal.dabrowski@ipipan.waw.pl, b.wojtas@nencki.gov.pl

Supplementary Information

Mutual Information

In this section, we define Mutual Information (*MI*), derived from Information Theory, which is a nonparametric measure of dependence between two variables. The main advantage of MI is that (unlike some classical measures of dependence like Pearson correlation) it is able to capture both linear and non-linear dependencies. In our analysis, we use *MI* to investigate dependencies between pairs of variables (e.g. gene expression vs survival) as well as to define Interaction Information that measures interaction's strength. We provide a definition of *MI* for discrete variables, however it is also possible to define MI for quantitative variables. Let *X* and *Y* be two variables and let $P(X = x)$ be the probability of observing outcome *x* for variable *X*. The basic quantity used in Information Theory is Entropy defined as

$$H(X) = -\sum_x P(X = x) \log P(X = x),$$

which quantifies the uncertainty present in the distribution of *X*. If the distribution is highly biased toward one particular value of *X*, then Entropy is low. If all values are equally likely, then *X* is maximal. The conditional entropy of *X* given *Y*

$$H(X|Y) = -\sum_y P(Y = y) \sum_x P(X = x|Y = y) \log P(X = x|Y = y)$$

measures uncertainty of the conditional distribution of *X* given *Y*. The Mutual Information is defined as

$$MI(X,Y) = H(X) - H(X|Y).$$

This can be interpreted as the amount of uncertainty in *X* which is removed by knowing *Y*, thus following the intuitive meaning of Mutual Information as the amount of information that one variable provides about another. If two variables are independent, then *MI* is zero. On the other hand, when there is a direct dependence between two variables, then *MI* attains its maximal value.

Analysis of interaction between Age/Gender and top 65 features

To analyze the interactions between the top 65 features (genes and methylations) and Age/Gender we use Interaction Information[1–3], which is a measure derived from Information Theory. It quantifies how strong is an interaction between two variables (in our case between Age/Gender and each of the top 65 features). The Interaction Information is defined as:

$$II(class, F, X) = MI(class, F|X) - MI(class, F),$$

where $class$ denotes survival (short or long), $F$is one of the top 65 features and X is age or gender. In addition, $MI(class, F)$ and $MI(class, F|X)$, in the formula, correspond to unconditional and conditional Mutual Information (MI), respectively; MI measures the dependence between two variables and is more general than a traditional correlation coefficient, since it is capable of detecting nonlinear dependencies. The conditional version of the Mutual Information measures the conditional dependence between F and $class$, given the additional variable X. So, the Interaction Information quantifies how much adding an additional variable X enhances the dependence between F and $class$. The Interaction Information may take positive values, negative values or zero. Positive values indicate that the conditional dependence is stronger than the unconditional one. The positive value also means that the dependence between F and $class$ may vary between different levels of X (for example it can be much stronger for the certain level of X than for the others). When the Interaction Information is around zero, we can conclude that X is redundant for explaining the relationship between F and $class$. Large negative values of II indicate that X inhibits the dependence between F and $class$.

The last two columns in Supplementary Table S2 show the values of II, given age and gender, respectively. It is seen that II is uniformly close to zero and therefore we can conclude that neither age nor gender affects the relationship between top 65 features and the survival significantly. This has been formally confirmed by a statistical test which has not rejected the null hypothesis $II = 0$ for all 65 pairs, given age, and separately, all 65 pairs, given gender. The above analysis proves that any of the conclusions pertaining to the relationships between survival time and methylations/genes is independent from both age and gender.

Additional features (molecular markers as well as clinical characteristics) derived from Ceccarelli et al. (2016)

List of additional features:

- 'Tissue.source.site'
- 'Study'
- 'BCR'
- 'Histology'
- 'Grade'
- 'Age'
- 'Gender'
- 'Karnofsky.Performance.Score'
- 'Mutation.Count'
- 'Percent.aneuploidy'
- 'IDH.status'
- 'X1p.19q.codeletion'
- 'IDH.codel.subtype'
- 'MGMT.promoter.status'
- 'Chr.7.gain.Chr.10.loss'
- 'Chr.19.20.co.gain'
- 'TERT.promoter.status'
- 'TERT.expression..log2.'
- 'TERT.expression.status'
- 'ATRX.status'
- 'DAXX.status'
- 'Telomere.Maintenance'
- 'BRAF.V600E.status'
- 'BRAF.KIAA1549.fusion'
- 'ABSOLUTE.purity'
- 'ABSOLUTE.ploidy'
- 'ESTIMATE.stromal.score'
- 'ESTIMATE.immune.score'
- 'ESTIMATE.combined.score'
- 'Original.Subtype'
- 'Transcriptome.Subtype'
- 'Pan.Glioma.RNA.Expression.Cluster'
- 'IDH.specific.RNA.Expression.Cluster'
- 'Pan.Glioma.DNA.Methylation.Cluster'
- 'IDH.specific.DNA.Methylation.Cluster'
- 'Supervised.DNA.Methylation.Cluster'

- 'Random.Forest.Sturm.Cluster'
- 'RPPA.cluster'
- 'Telomere.length.estimate.in.blood.normal..Kb.'
- 'Telomere.length.estimate.in.tumor..Kb.'

Link to the original data: http://ars.els-cdn.com/content/image/1-s2.0-S009286741501692X-mmc2.xlsx

Structure prediction REST-DNA complex

The structure of the REST protein was predicted using template-based modeling protocol in **I**terative **T**hreading **ASSE**mbly **R**efinement (I-TASSER) server[4]. Five models were obtained from the modeling, which were built using homologous zinc finger proteins (e.g. PDB codes: 3GAV, 1TF6). Good quality model 1 from I-TASSER prediction, which has the highest C-score value (estimated confidence of predicted models) and highest number of decoys, was selected for further analysis (Supplementary Table S5). Moreover, analysis of sequentially similar proteins with known structures, performed by PSI-BLAST (Position-Specific Iterated BLAST, version BLASTP 2.6.1+)[5], showed that the N-terminal fragment of REST resembles high similarity to other proteins, which includes amino-acids residues from ~150 to ~430 which confirms the high reliability of obtained structure. In the next step, predicted model of the REST protein, restricted to the N-terminal domain due to size-limitations of the method, was docked to the B-DNA model of the DNA binding motif using NPdock server[6]. N-terminal part of the REST protein was chosen also because of the literature data, which suggest that DNA is binding to the N-terminal part of the protein[7] and because only this part of REST protein was predicted with high accuracy. After rigid docking done by NPdock server, side-chains of REST were optimized using rotamer database search[8] to avoid clashes and subsequently whole system was energy minimized. Then system was subjected to the short all-atom molecular dynamics simulation to relax the structure and optimize contacts between REST and DNA using YASARA software[9].

Multiple Survival Screening (MSS)

In order to additionally validate our results we use Multiple Survival Screening (MSS) algorithm[10]. The main goal of the method is to ensure robustness of the chosen subset of features. We briefly recall the idea of MSS. The method involves generating random sets of observations (so-called Random Datasets - RDSs) and random sets of features (Random Gene Sets - RGSs). For each combination of RDS and RGS, a clustering algorithm is used to determine two homogeneous groups of observations. Next, the log-rank test is used to verify whether the survival curves differ between the two clusters. The method assigns a p-value to each random set of features (RGS); the smaller the p-value, the more significant is a given feature set.

To confirm significance of the features found in our analysis (using MCFS algorithm) we carried out an additional experiment using MSS. We used the parameters recommended by Li et. al.[10]. In particular, we generated 10^5 distinct random feature sets by randomly selecting 30 features and 74 random sets of observations. We ran the algorithm in three scenarios. In the first experiment, we applied MSS on the top 65 features selected by the MCFS algorithm. Figure S11 shows a histogram of p-values corresponding to random subsets of top 65 features chosen by the MCFS algorithm. Each random subset contains 30 features. Observe that all p-values are very small, which additionally confirms the relevance of the features selected by the MCFS. In the second experiment, we ran MSS on the set of remaining features, i.e. the complement of the set of top 65 features chosen by the MCFS. Figure S12 shows a histogram of the p-values corresponding to the second experiment. Note that in this case p-values are larger than in the previous case. In addition, we performed an experiment in which values of the top 65 features selected by the MCFS are randomly permuted. Random rearrangement of the feature values makes them not significant (i.e. they do not affect the survival time any more) and having marginal distributions fixed. This experiment gives an insight into the nature of the p-vales for spurious features. Figures S13 shows a histogram of the p-values corresponding to the third experiment.

Both methods, MCFS and MSS, use Monte Carlo approach to run analysis on randomly selected subsets of features. However, MCFS builds thousands of decision trees (or regression trees) that select only predictive features into the model. Relative Importance measure takes into account goodness of split at each tree node, number of objects split at (by) the node (feature) and the tree's overall prediction quality on a testing set. If a feature is frequently selected to build the tree model and plays significant role within that tree it is considered as important. Notice that a feature can frequently occur in random subsets and still be almost always ignored during tree model building because of its lack of predictive ability. Thus, its RI proves, correctly, very low (or zero). In contrast, MSS evaluates highly a whole random subset of features if any of them has high predictive ability. This leads to high evaluation of not significant but lucky features that occurred in the subset along with the important one.

Supplementary Figures

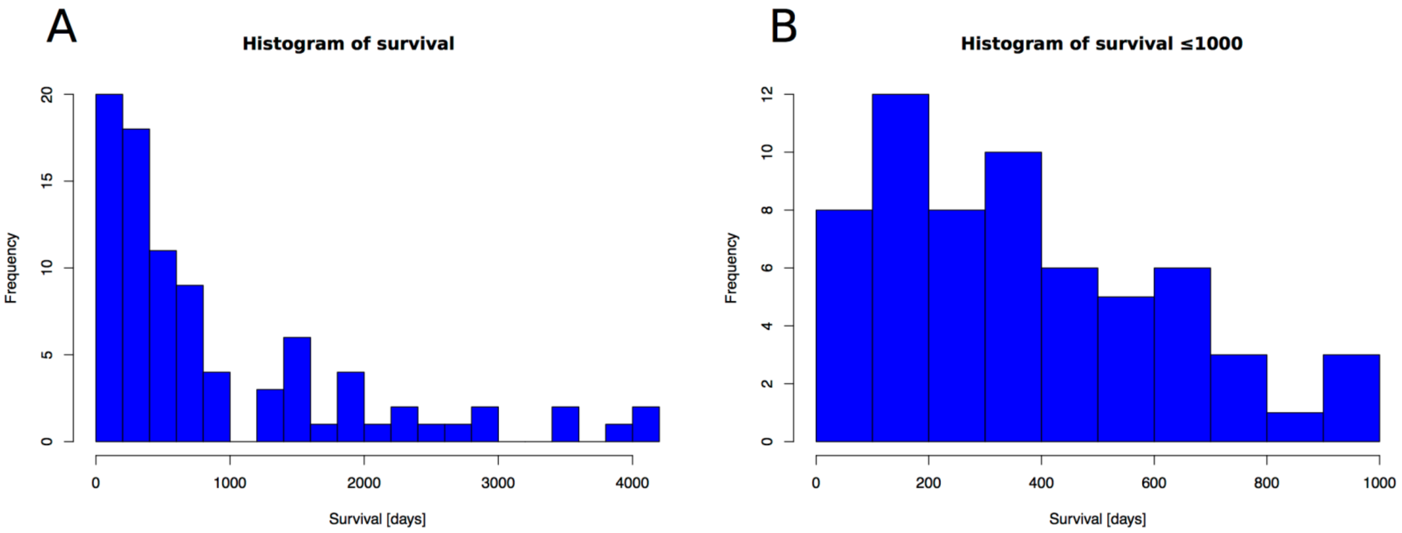

Supplementary Figure S1. Histogram of overall survival of the 88 patients included in this study. (**A**) Overall survival among all patients ranged from 7 to 4084 days. (**B**) Overall survival histogram drops above 400 days.

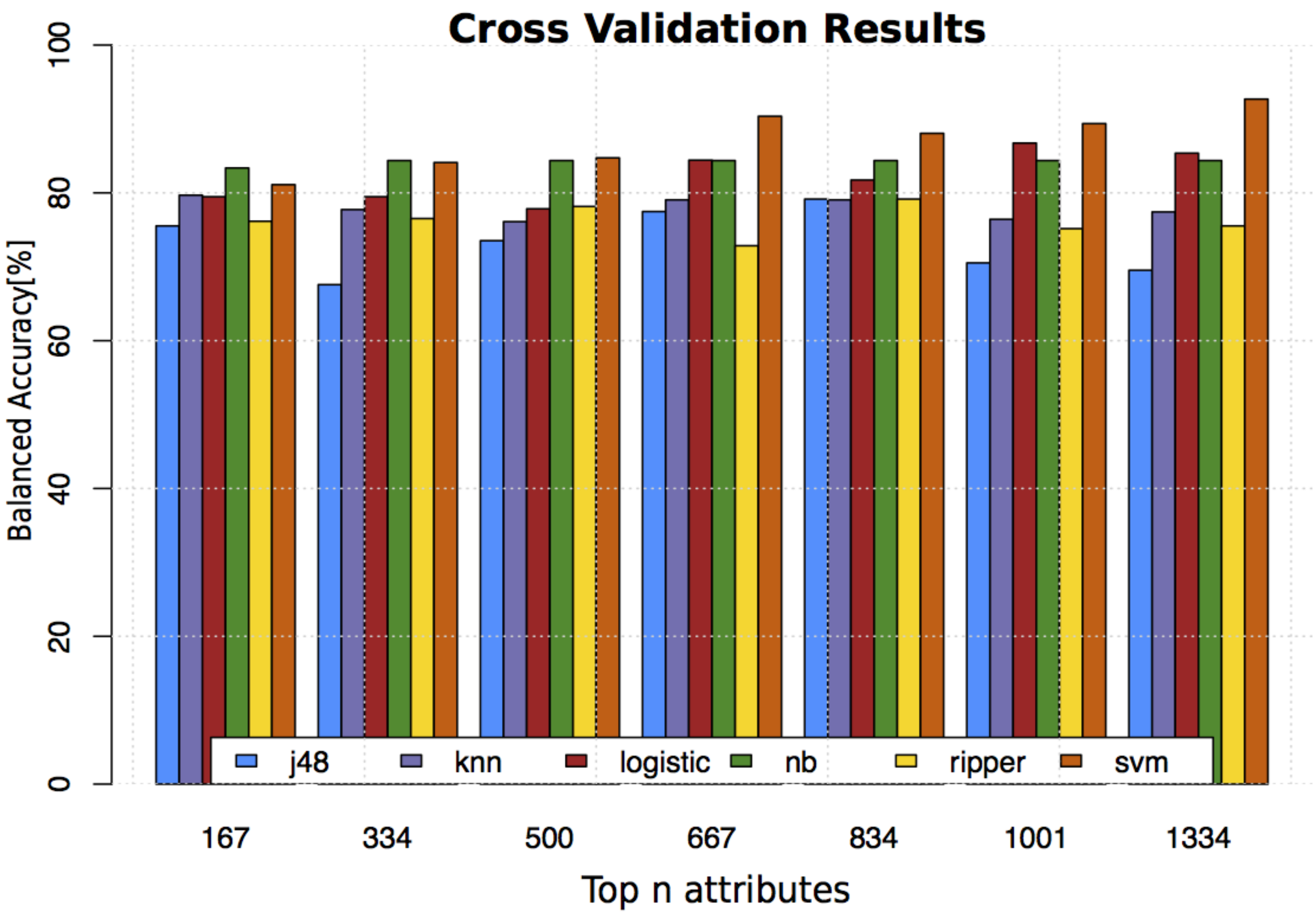

Supplementary Figure S2. Balanced accuracy of the cross-validation of various algorithms and sets of features. On the basis of the various numbers of significant features, patients were assigned to the correct decision class using various classifiers. For details about balanced accuracy see for example Broderson et al. (2010); j48 - decision tree, knn - k Nearest Neighbours, logistic - logistic regression, nb - naive Bayes, ripper - rule induction algorithm, svm - support vector machines.

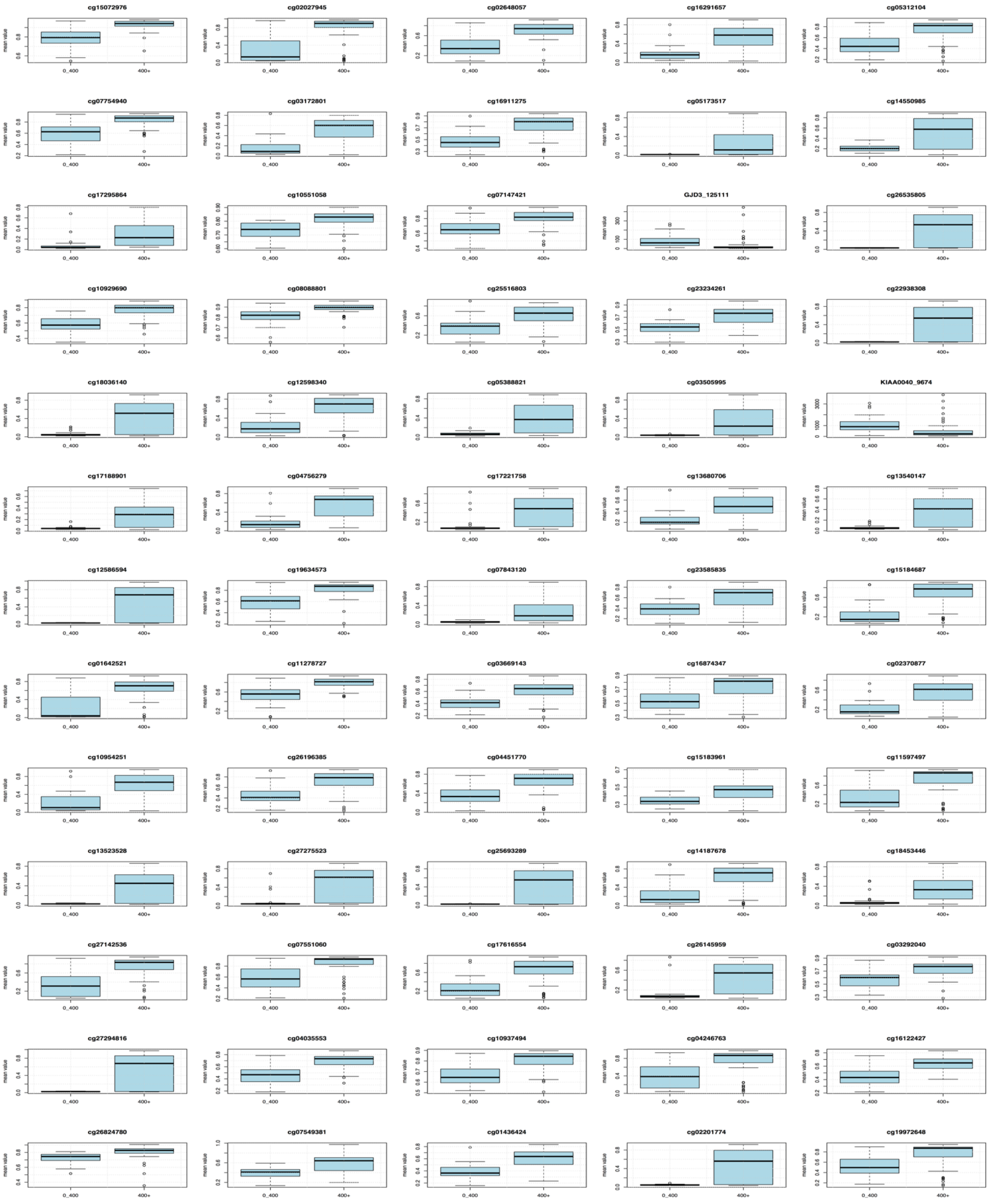

Supplementary Figure S3. Pairwise comparison of each feature using boxplots, showing the distribution of the significant features between the two decision classes. There were significant differences between *≤400* and *400+* for all 65 significant features (Kruskal-Wallis test with Bonferroni correction). The level of p-values ranged from 10e-06 to 10e-10 and was so small regarding the number of features, that even strict Bonferroni correction kept all differences statistically significant.

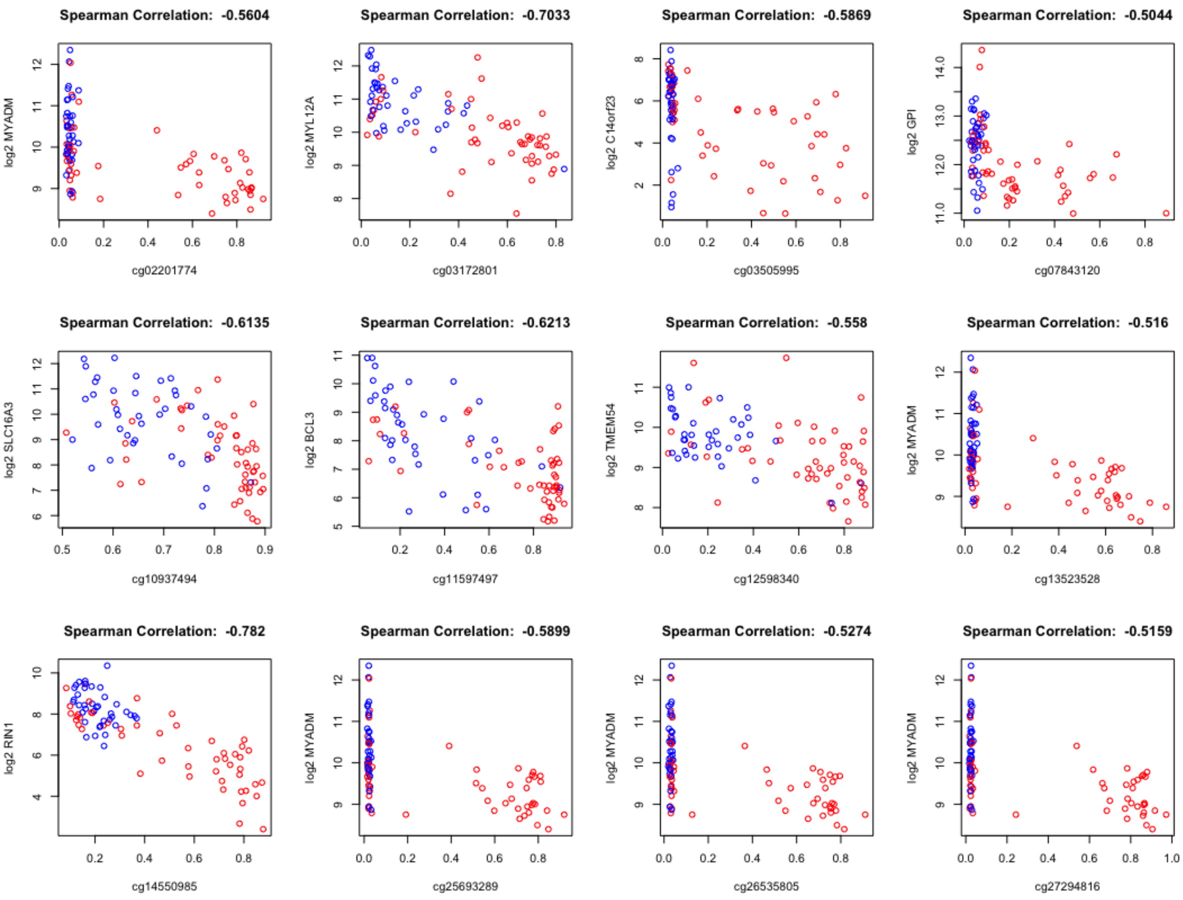

Supplementary Figure S4. The strongest correlations (abs(Spearman Correlation)>0.5) between methylations β-values and corresponding them gene expression levels.

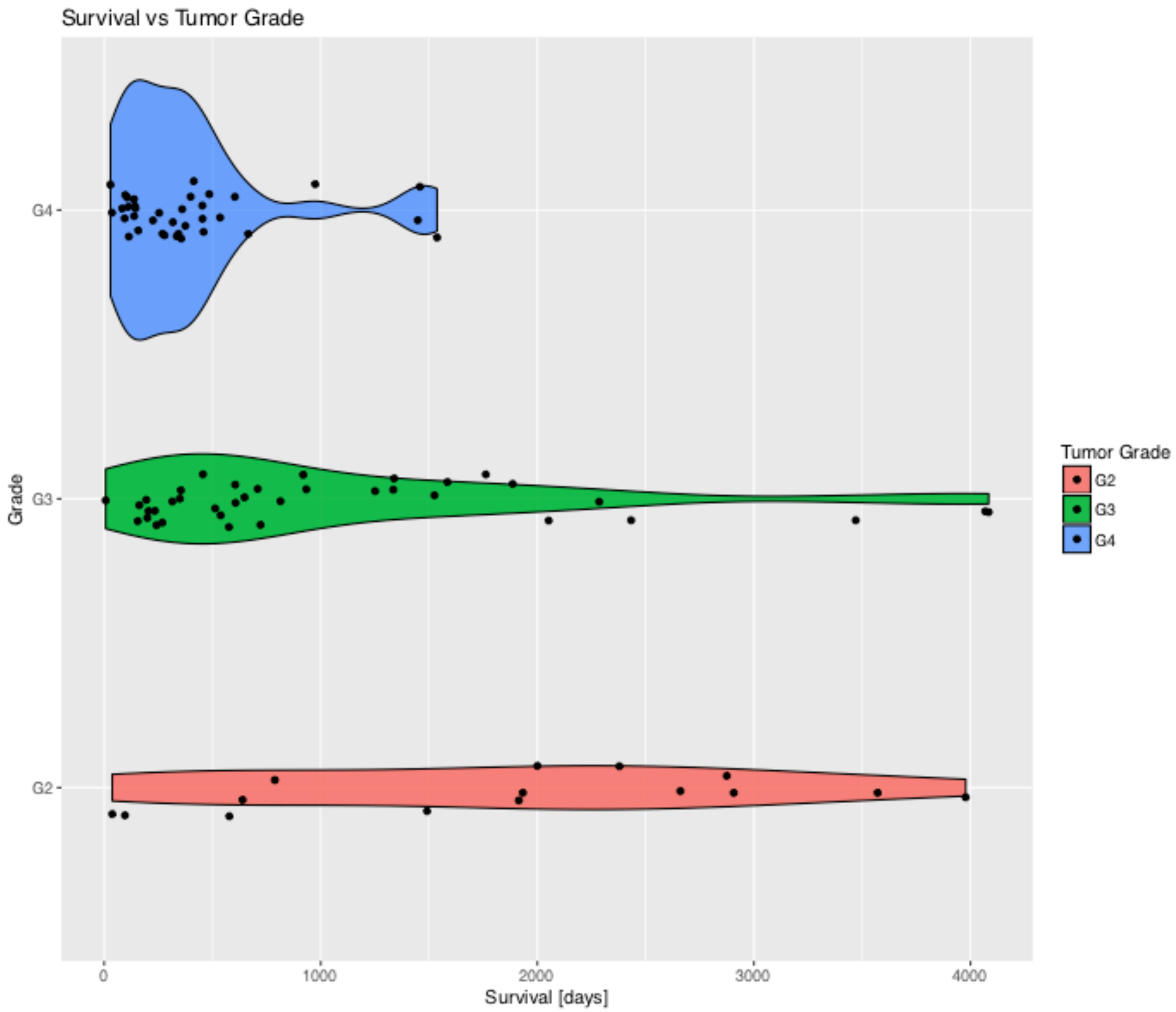

Supplementary Figure S5. Violin plots of patient's survival within tumors of different grades.

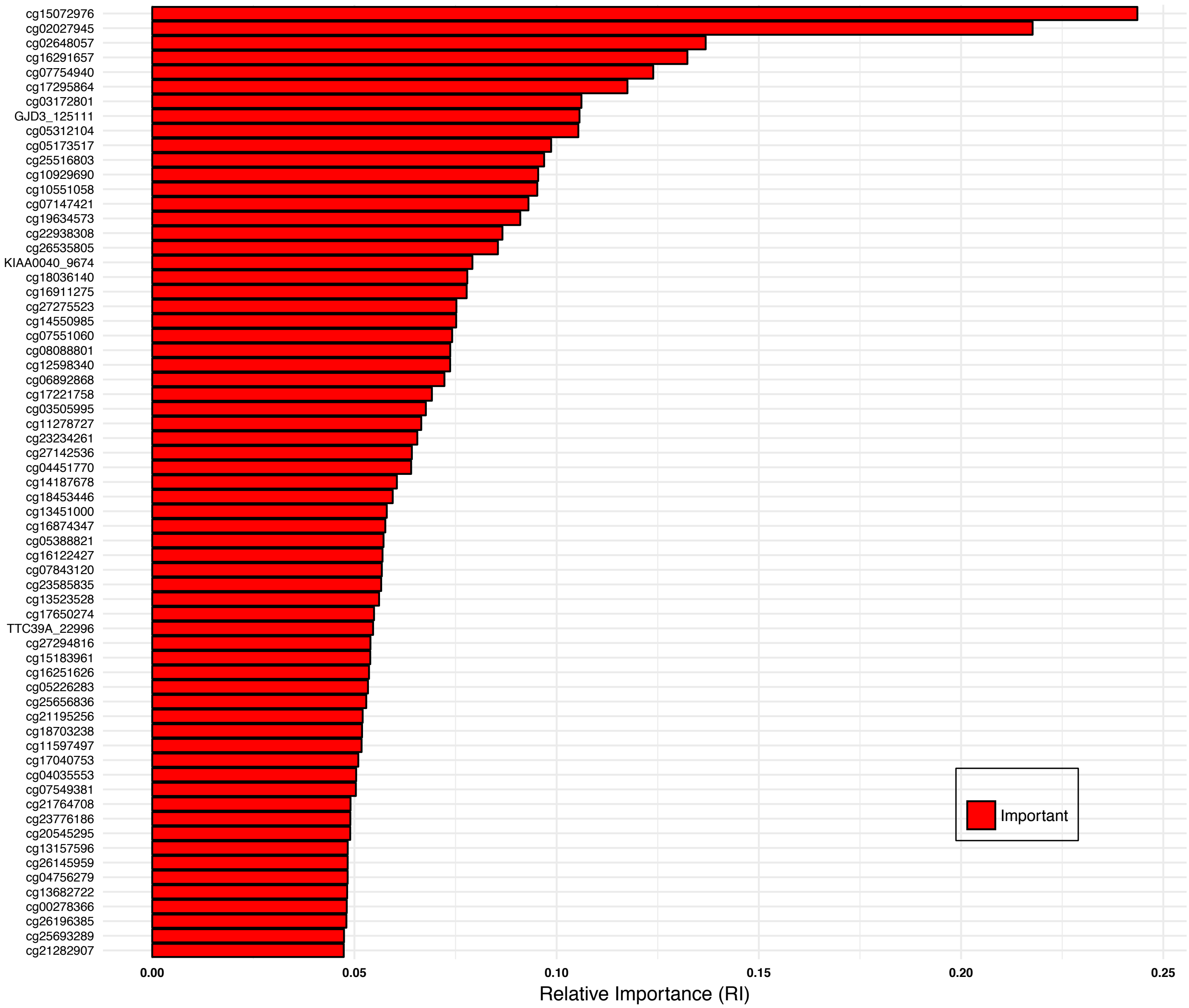

Supplementary Figure S6. Top 65 features obtained from the second MCFS-ID analysis. It was performed on the top 5k features from the first MCFS-ID analysis mixed with patients' characteristics taken from Ceccarelli et al. (2016).

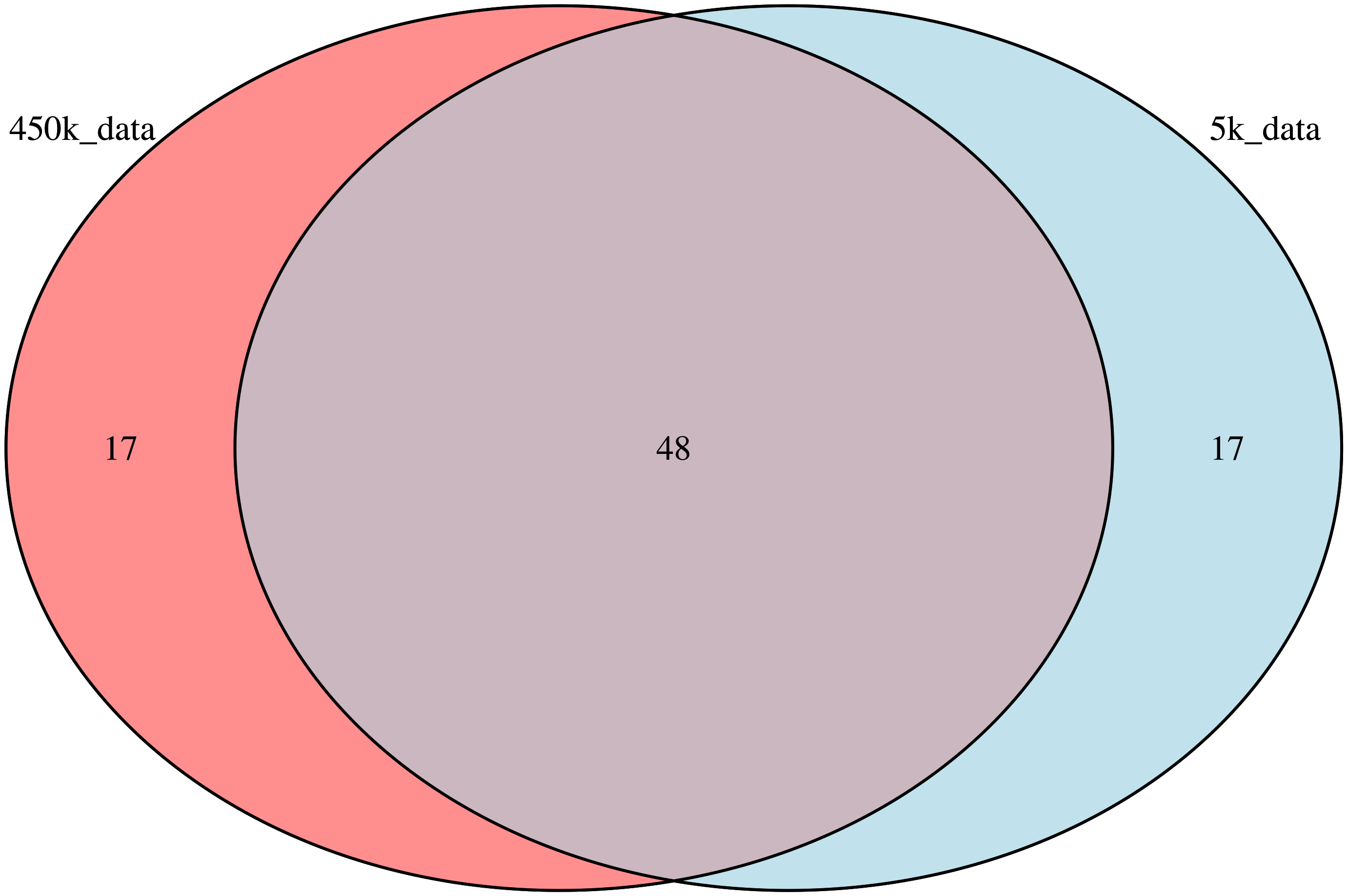

Supplementary Figure S7. The overlap between the top 65 features from the rankings obtained from the first and the second MCFS-ID analysis (first performed on 450k input features and the second on top 5k features obtained from the first MCFS-ID analysis mixed with patients' characteristics).

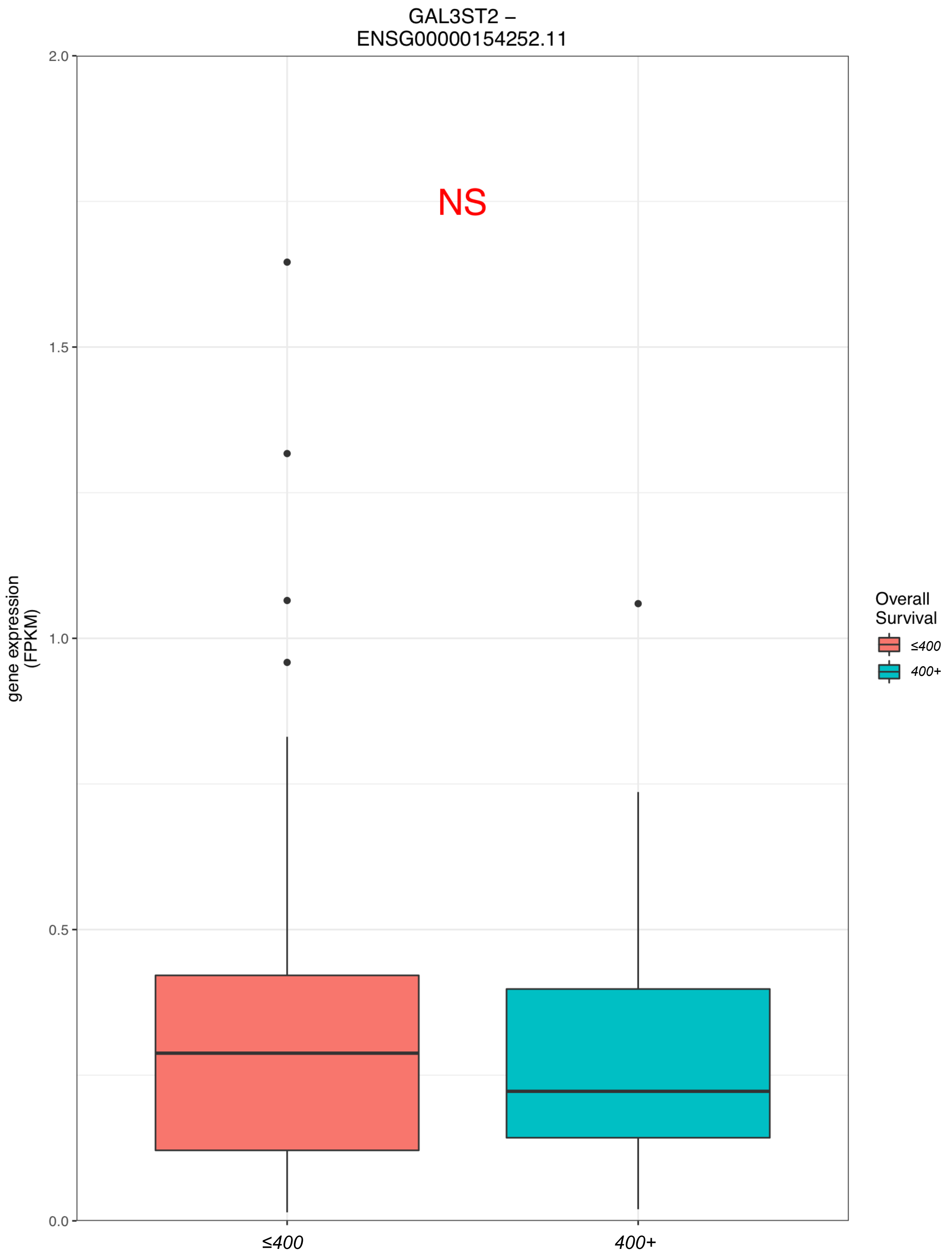

Supplementary Figure S8. Expression level of the gene *GAL3ST2* that is located downstreams the top methylation site cg15072976, as predicted by MCFS. Here we are comparing the expression of the gene in the ≤400 (pink) and 400+ (blue) days survival groups. The label "NS" in between the two boxplots stands for "No Significance", since there is no statistically significant change in the gene expression.

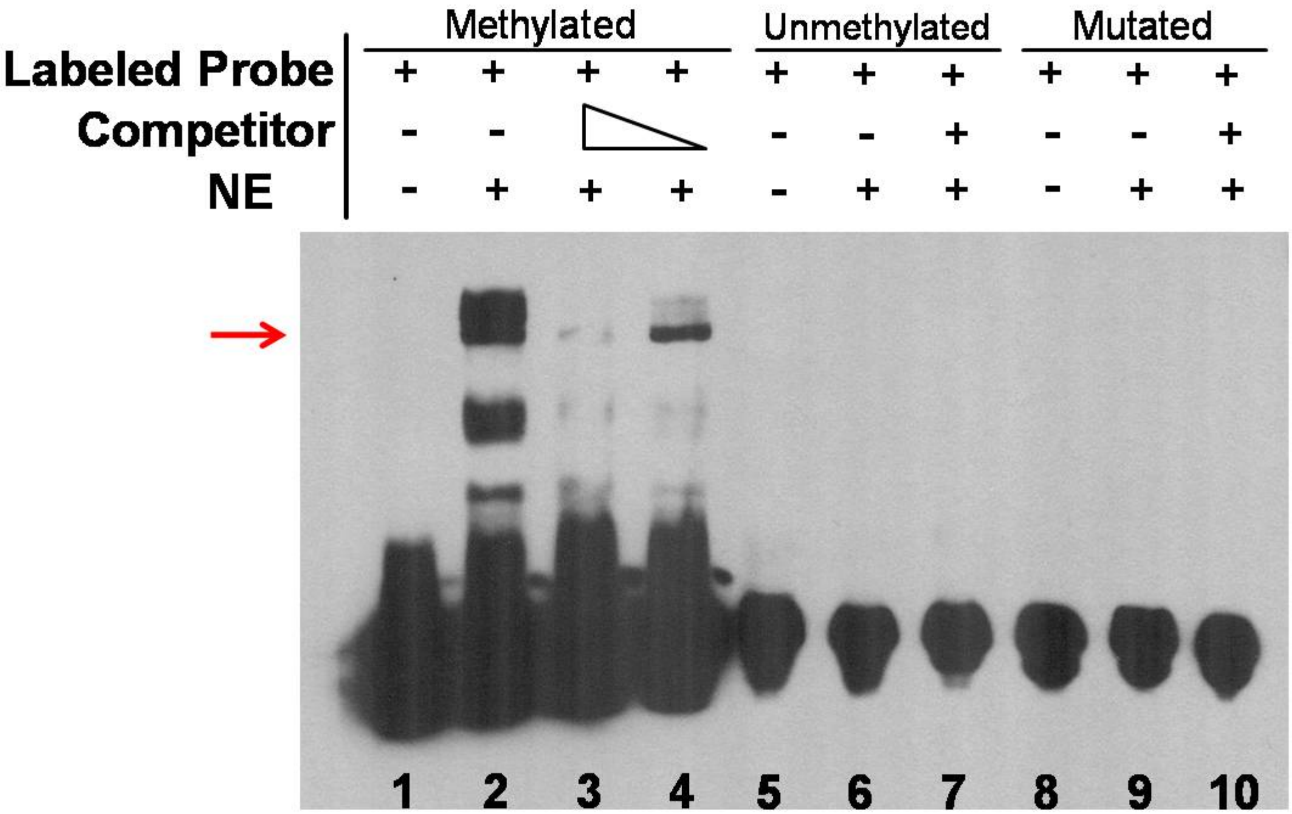

Supplementary Figure S9. Electrophoretic mobility shift assay showing protein binds to DNA sequence containing REST consensus motif with methylated CpG site identified as a top feature by MCFS-ID (cg15072976). The experiment performed for LN18 glioma cell line. Three variants of biotin labeled DNA probe containing REST consensus motif were used in the experiment: methylated or unmethylated CpG site or a probe carrying CG→AT nucleotide substitution. Competition assays were performed using a corresponding unlabeled probe (competitor). For the methylated probe 2 variants of differing molar concentration of the competitor from high to low were used. The control lanes with no nuclear extract (NE) added (lane 1, 5 and 8) indicate band pattern that is given by a free probe.

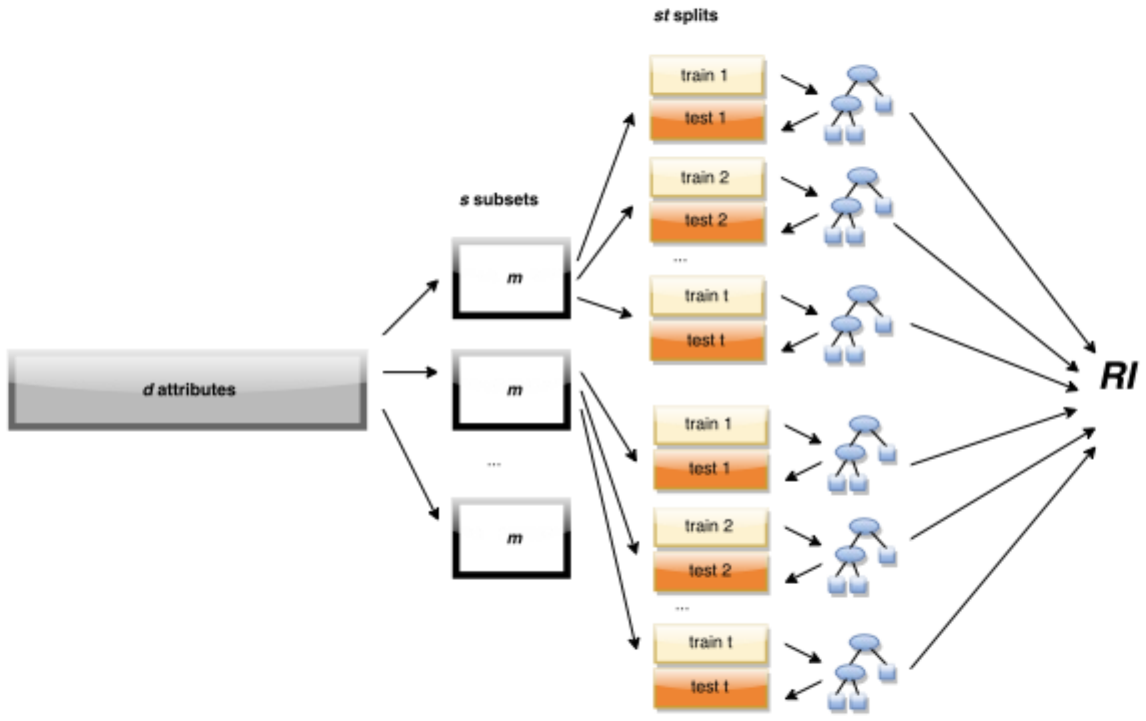

Supplementary Figure S10. Block diagram of the main steps of the MCFS-ID pipeline procedure.

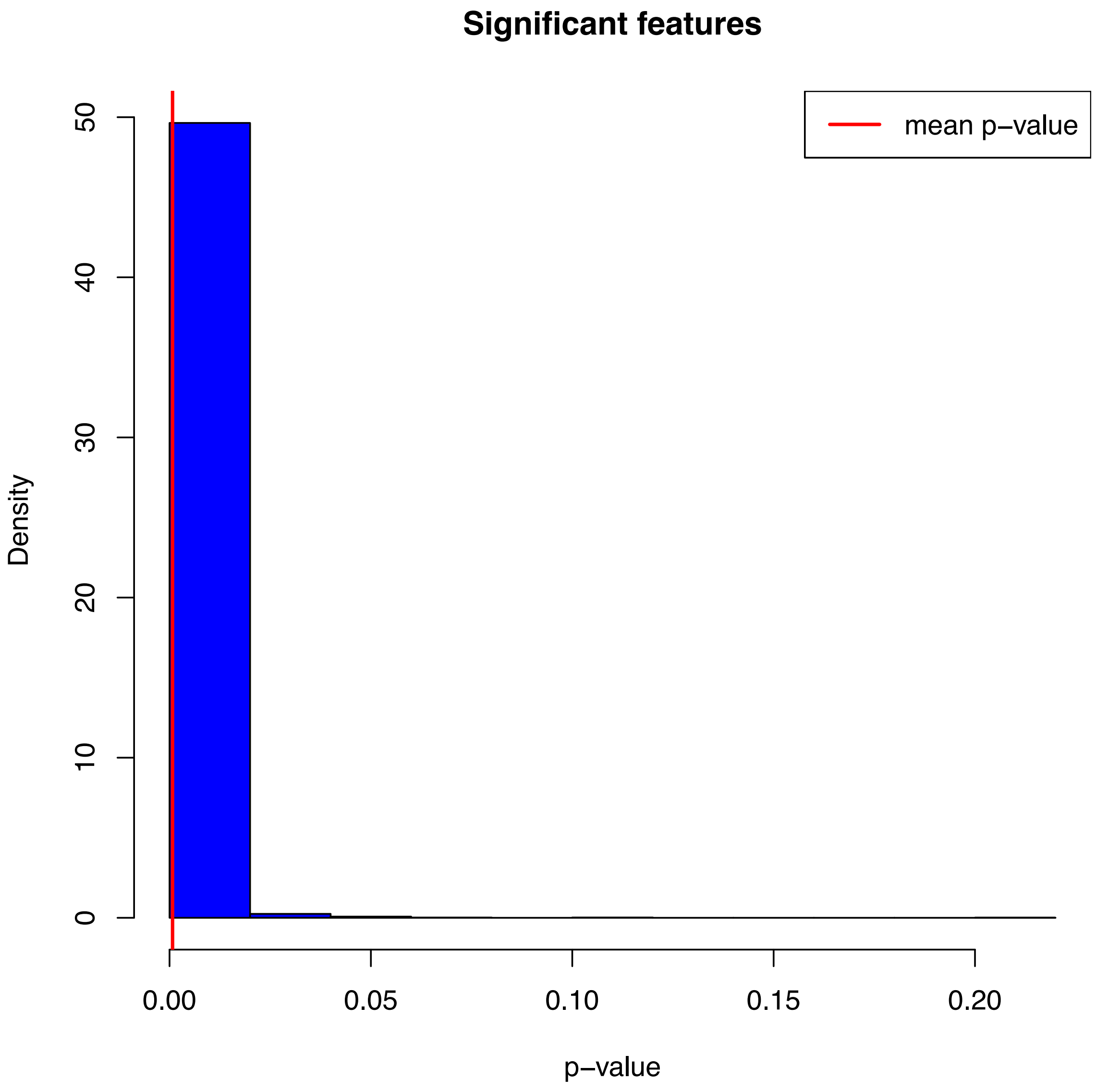

Supplementary Figure S11. Histogram of p-values corresponding to random subsets of top 65 features chosen by MCFS algorithm. Each random subset contains 30 features. The p-values are calculated using MSS algorithm.

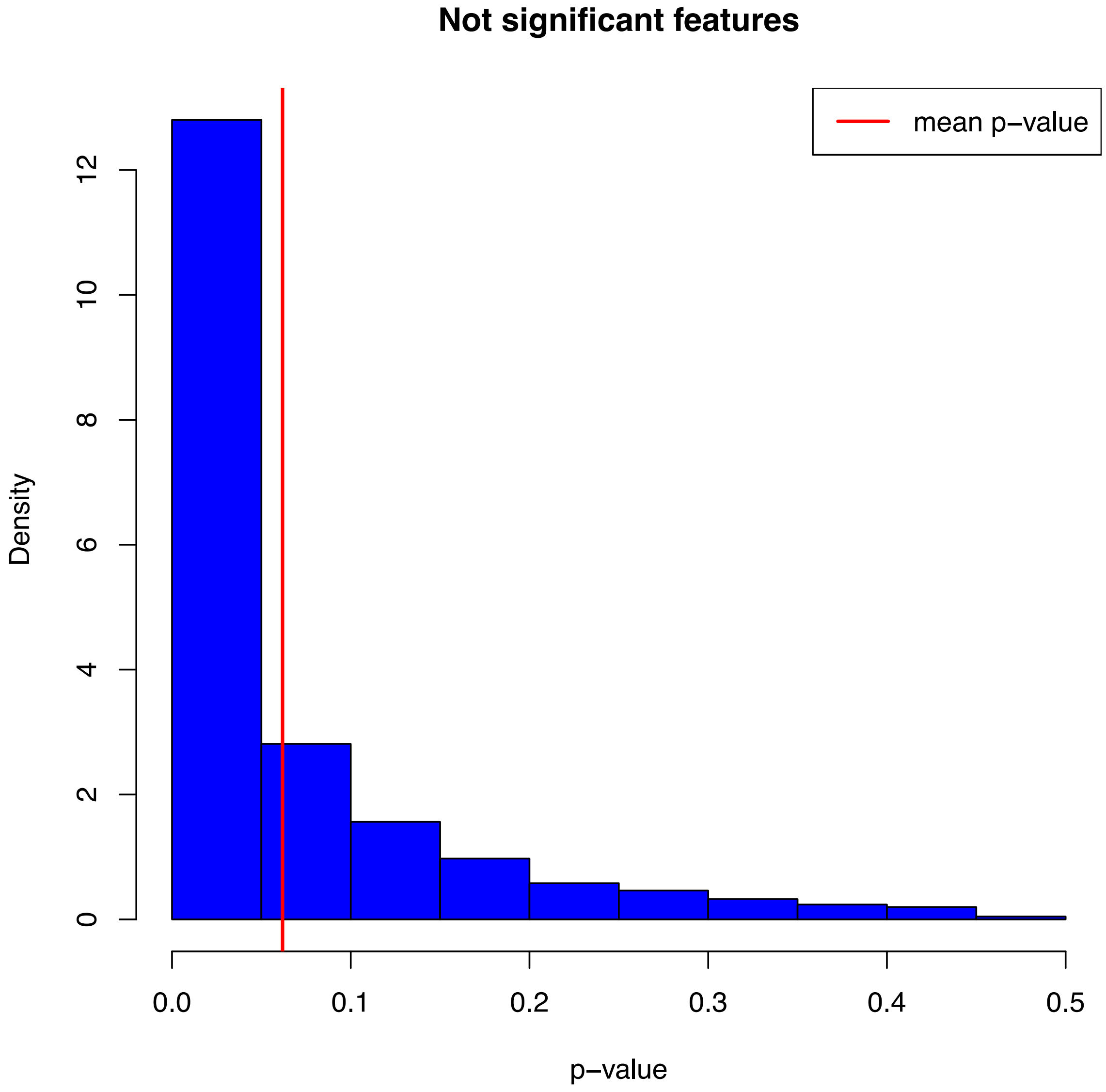

Supplementary Figure S12. Histogram of p-values corresponding to random subsets of the complement of the set of top 65 features chosen by MCFS. Each random subset contains 30 features. The p-values are calculated using MSS algorithm.

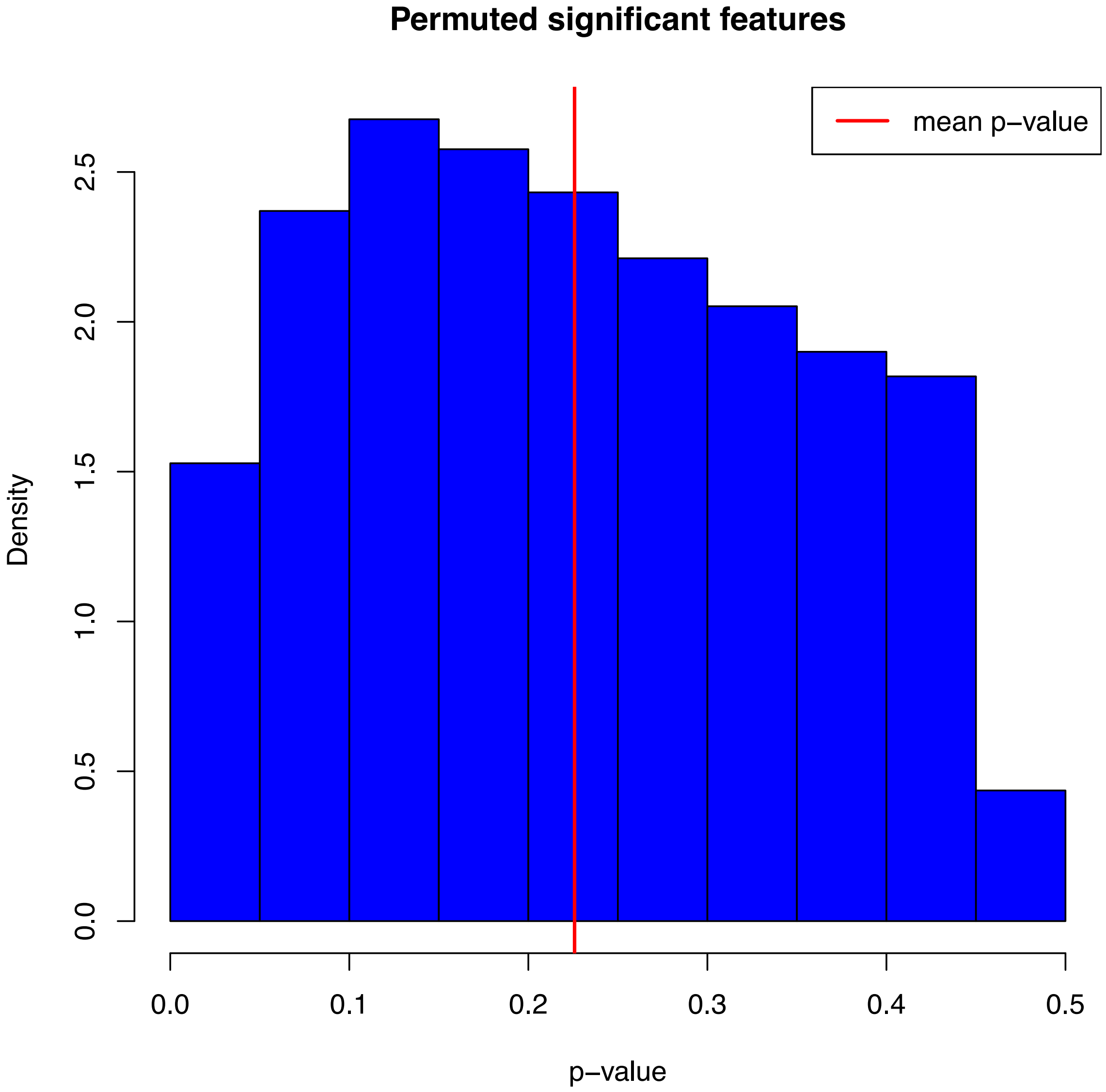

Supplementary Figure S13. Histogram of p-values corresponding to random subsets of top 65 features chosen by MCFS algorithm, with randomly permuted values. Each random subset contains 30 features. The p-values are calculated using MSS algorithm.

Supplementary Tables

Supplementary Table S1. The molecular and clinical characteristics of the 88 patients included in the short and long overall survival organized in the decision table, where "Days to death" feature was changed into the "Decision". When running MCFS-ID columns No, Patient ID, Days to death were removed.

No.	Patient ID	Beta values of DNA methylation sites			RNA-seq expression levels			Clinical records			Days to death	Decision
		Site 1	…	Site 396065	Expression 1	…	Expression 19943	Grade	Gender	Age		
		cg00000029	…	rs9839873	A1BG	…	TAKR					
1	TCGA-CS-4941-01	0,655	…	0,941	72,233	…	0,000	G3	MALE	67	234	"≤400"
2	TCGA-CS-4942-01	0,826	…	0,067	74,453	…	0,000	G3	FEMALE	44	1335	"400+"
3	TCGA-DU-6406-01	0,179	…	0,042	19,516	…	0,000	G2	MALE	43	639	"400+"
…	…	…	…	…	…	…	…	…	…	…	…	…
88	TCGA-06-5411-01	0,540	…	0,939	293,241	…	0,000	G4	MALE	51	254	"≤400"

Supplementary Table 2. Relative Importance (RI), Mutual Information (MI) and Interaction Information (II) computed for the significant features returned by MCFS-ID.

Feature	RI	MI (feature, survival)	II (feature, survival, age)	II (feature, survival, gender)
cg15072976	0,303	0.264 *	-0.064	0.005
cg02027945	0,275	0.306 *	-0.088	0.002
cg02648057	0,217	0.226 *	-0.02	0.006
cg16291657	0,215	0.264 *	-0.079	0.003
cg05312104	0,209	0.226 *	-0.067	0.002
cg07754940	0,208	0.264 *	-0.045	0
cg03172801	0,186	0.264 *	-0.045	0
cg16911275	0,178	0.264 *	-0.051	0.003
cg05173517	0,176	0.226 *	-0.067	0.008
cg14550985	0,176	0.11	-0.004	0.004
cg17295864	0,175	0.134	-0.049	0.001
cg10551058	0,174	0.192 *	-0.05	0.007
cg07147421	0,172	0.306 *	-0.059	0.005
GJD3_125111	0,17	0.264 *	-0.081	0.006
cg26535805	0,169	0.161 *	-0.055	0.009
cg10929690	0,167	0.226 *	-0.081	0.011
cg08088801	0,166	0.264 *	-0.073	0.027
cg25516803	0,165	0.161 *	-0.048	0.012
cg23234261	0,165	0.192 *	-0.05	0.003
cg22938308	0,165	0.088	-0.04	0.003
cg18036140	0,162	0.11	-0.052	0.004
cg12598340	0,158	0.264 *	-0.034	0
cg05388821	0,148	0.161 *	-0.06	0.005
cg03505995	0,147	0.161 *	-0.048	0.009
KIAA0040_9674	0,147	0.192 *	-0.053	0.002
cg17188901	0,147	0.226 *	-0.067	0.013
cg04756279	0,146	0.226 *	-0.064	0
cg17221758	0,145	0.192 *	-0.015	0.002
cg13680706	0,143	0.226 *	-0.073	0.008
cg13540147	0,142	0.161 *	-0.035	0.025
cg12586594	0,14	0.134	-0.047	0.012
cg19634573	0,138	0.226 *	-0.009	0.002
cg07843120	0,137	0.192 *	-0.052	0.003
cg23585835	0,137	0.161 *	-0.039	0.001
cg15184687	0,137	0.226 *	-0.059	0.009
cg01642521	0,135	0.226 *	-0.064	0.001
cg11278727	0,135	0.226 *	-0.059	0
cg03669143	0,134	0.226 *	-0.067	0
cg16874347	0,133	0.161 *	-0.039	0.008
cg02370877	0,132	0.226 *	-0.029	0.002
cg10954251	0,131	0.192 *	-0.052	0.003
cg26196385	0,131	0.264 *	-0.045	0
cg04451770	0,13	0.192 *	-0.012	0.002
cg15183961	0,13	0.134	0.019	0.002
cg11597497	0,129	0.226 *	-0.064	0.001
cg13523528	0,128	0.192 *	-0.068	0.002
cg27275523	0,127	0.226 *	-0.067	0.013
cg25693289	0,126	0.088	-0.025	0.02
cg14187678	0,126	0.226 *	-0.065	0.009
cg18453446	0,126	0.226 *	-0.059	0.002
cg27142536	0,126	0.264 *	-0.042	0
cg07551060	0,125	0.264 *	-0.045	0.011
cg17616554	0,124	0.226 *	-0.029	0
cg26145959	0,124	0.192 *	-0.015	0.002
cg03292040	0,124	0.161 *	-0.039	0.001
cg27294816	0,124	0.11	-0.032	0.001
cg04035553	0,123	0.192 *	-0.015	0.008
cg10937494	0,123	0.161 *	-0.048	0.005
cg04246763	0,122	0.192 *	-0.05	0.003
cg16122427	0,121	0.161 *	-0.005	0.004
cg26824780	0,121	0.192 *	-0.085	0.022
cg07549381	0,121	0.11	-0.043	0.001
cg01436424	0,121	0.192 *	-0.045	0.007
cg02201774	0,118	0.134	-0.065	0.005

cg19972648	0,118	0.192 *	-0.052	0.002

* marks significant results

Supplementary Table S3. The order of the sense and antisense oligonucleotide annealing former to EMSA experiment.

probe	sense oligonucleotide	antisense oligonucleotide
**REST_me-dC_bio**<br><br>**(labeled and methylated probe)**	bio-ACTCATGG(5-Me-dC)GCGTGGCCCGTAGCTCTGAGGAGCA	TGCTCCTCAGAGCTACGGGCCACG(5-Me-dC)GCCATGAGT
**REST_dC_bio**<br><br>**(labled and unmetylated probe)**	bio-ACTCATGGCGCGTGGCCCGTAGCTCTGAGGAGCA	TGCTCCTCAGAGCTACGGGCCACGCGCCATGAGT
**REST_mut_bio**<br><br>**(labeled and mutated probe)**	bio-ACTCATGG*AT*CGTGGCCCGTAGCTCTGAGGAGCA	TGCTCCTCAGAGCTACGGGCCACG*AT*CCATGAGT
**REST_me-dC**<br><br>**(methylated competitor)**	ACTCATGG(5-Me-dC)GCGTGGCCCGTAGCTCTGAGGAGCA	TGCTCCTCAGAGCTACGGGCCACG(5-Me-dC)GCCATGAGT
**REST_dC**<br><br>**(unmethyled competitor)**	ACTCATGGCGCGTGGCCCGTAGCTCTGAGGAGCA	TGCTCCTCAGAGCTACGGGCCACGCGCCATGAGT
**REST_mut (mutated competitor)**	ACTCATGG*AT*CGTGGCCCGTAGCTCTGAGGAGCA	TGCTCCTCAGAGCTACGGGCCACG*AT*CCATGAGT

me=methyl group

bio=biotin

Supplementary Table S4. ChIP-seq experiments for the five glioma-related cell lines

Cell Line	NCBI Study Alias	ChIP-seq
Gliobla	GSE33213	CTCF
Gliobla	GSE33213	CTCF
Gliobla	GSE33213	Pol2
Gliobla	GSE33213	Pol2
Gliobla	GSE33213	Input
T98G	GSE72476	H3K9ac
T98G	GSE72476	Input
T98G	GSE72476	RBBP4
T98G	GSE72476	Input
U87	GSE32465	Input
U87	GSE32465	Input
U87	GSE32465	Input
U87	GSE32465	Input
U87	GSE32465	Pol2
U87	GSE32465	Pol2
U87	GSE32465	Pol2
U87	GSE32465	Pol2
U87	GSE32465	Input
U87	GSE32465	Input
U87	GSE32465	Input
U87	GSE32465	Input
U87	GSE32465	NRSF
U87	GSE32465	NRSF
U87	GSE32465	NRSF
U87	GSE32465	NRSF
BE2-C	GSE30263	CTCF
BE2-C	GSE30263	CTCF
BE2-C	GSE30263	Input
BE2-C	GSE30263	Input
BE2-C	GSE30263	CTCF
HA-sp	GSE30263	CTCF
HA-sp	GSE30263	Input
BE2-C	GSE35583	H3K4me3
BE2-C	GSE35583	H3K4me3
BE2-C	GSE35583	Input
BE2-C	GSE35583	Input
HA-sp	GSE35583	H3K4me3
HA-sp	GSE35583	H3K4me3
HA-sp	GSE35583	Input

Supplementary Table S5. C-score values of the models from I-TASSER[4,11].

Model no.	C-score[a]	No.of decoys[b]	Cluster density[c]
**1**	-0,63	363	0.1782
**2**	-3,10	62	0.0150
**3**	-3,17	50	0.0140
**4**	-3,27	49	0.0127
**5**	-3,30	49	0.0123

[a] C-score is a confidence score for estimating the quality of the predicted models.

[b] Number of replicas (structures) in each model.

[c] Number of structures present in a given cluster. Higher cluster density means that the structure appears more often in the simulation trajectory and has a better-quality model.

Supplementary Table S6. Detailed description about clinical characteristics of the 88 patients included in MCFS analysis.

ID	Tissue.source.site	Study	Histology	Grade	Age	Gender	IDH.status	X1p.19q.codeletion	IDH.codel.subtype	MGMT.promoter.status	Chr.7.gain.Chr.10.loss	Chr.19.20.co.gain	TERT.promoter.status	Telomere.Maintenance	decision
TCGA-CS-4941-01	Thomas_Jefferson_University	Brain_Lower_Grade_Glioma	astrocytoma	G3	67	male	WT	non_codel	IDHwt	Methylated	Gain_chr_7_&_loss_chr_10	No_chr_19_20_gain	Mutant	TERT	0_400
TCGA-CS-4942-01	Thomas_Jefferson_University	Brain_Lower_Grade_Glioma	astrocytoma	G3	44	female	Mutant	non_codel	IDHmut_non_codel	Unmethylated	No_combined_CNA	No_chr_19_20_gain	WT	ATRX	400+
TCGA-CS-5395-01	Thomas_Jefferson_University	Brain_Lower_Grade_Glioma	oligodendroglioma	G2	43	male	WT	non_codel	IDHwt	Unmethylated	Gain_chr_7_&_loss_chr_10	Gain_chr_19_20	Mutant	TERT	400+
TCGA-CS-5397-01	Thomas_Jefferson_University	Brain_Lower_Grade_Glioma	astrocytoma	G3	54	female	WT	non_codel	IDHwt	Unmethylated	Gain_chr_7_&_loss_chr_10	No_chr_19_20_gain	Mutant	TERT	0_400
TCGA-CS-6186-01	Thomas_Jefferson_University	Brain_Lower_Grade_Glioma	oligoastrocytoma	G3	58	male	WT	non_codel	IDHwt	Unmethylated	Gain_chr_7_&_loss_chr_10	No_chr_19_20_gain	Mutant	TERT	400+
TCGA-DH-5140-01	University_of_Florida	Brain_Lower_Grade_Glioma	oligoastrocytoma	G3	38	female	WT	non_codel	IDHwt	Unmethylated	No_combined_CNA	No_chr_19_20_gain	WT		400+
TCGA-DH-A669-02	University_of_Florida	Brain_Lower_Grade_Glioma	oligodendroglioma	G3	70	male	Mutant	codel	IDHmut_codel	Methylated	No_combined_CNA	No_chr_19_20_gain	Mutant	TERT	400+
TCGA-DU-5852-01	Henry_Ford_Hospital	Brain_Lower_Grade_Glioma	oligoastrocytoma	G3	61	female	WT	non_codel	IDHwt	Methylated	No_combined_CNA	No_chr_19_20_gain	Mutant	NA	0_400
TCGA-DU-6393-01	Henry_Ford_Hospital	Brain_Lower_Grade_Glioma	oligodendroglioma	G3	66	male	Mutant	codel	IDHmut_codel	Methylated	No_combined_CNA	No_chr_19_20_gain	Mutant	TERT	400+
TCGA-DU-6395-01	Henry_Ford_Hospital	Brain_Lower_Grade_Glioma	oligoastrocytoma	G2	31	male	Mutant	non_codel	IDHmut_non_codel	Methylated	No_combined_CNA	No_chr_19_20_gain	NA	NA	400+
TCGA-DU-6396-01	Henry_Ford_Hospital	Brain_Lower_Grade_Glioma	oligoastrocytoma	G3	31	female	Mutant	non_codel	IDHmut_non_codel	Methylated	No_combined_CNA	No_chr_19_20_gain	WT	ATRX	400+
TCGA-DU-6399-01	Henry_Ford_Hospital	Brain_Lower_Grade_Glioma	oligodendroglioma	G2	54	male	Mutant	non_codel	IDHmut_non_codel	Methylated	No_combined_CNA	No_chr_19_20_gain	WT	ATRX	400+
TCGA-DU-6400-01	Henry_Ford_Hospital	Brain_Lower_Grade_Glioma	oligodendroglioma	G2	66	female	Mutant	codel	IDHmut_codel	Methylated	No_combined_CNA	No_chr_19_20_gain	Mutant	TERT	0_400
TCGA-DU-6401-01	Henry_Ford_Hospital	Brain_Lower_Grade_Glioma	oligodendroglioma	G2	31	female	Mutant	non_codel	IDHmut_non_codel	Methylated	No_combined_CNA	No_chr_19_20_gain	WT	ATRX	400+
TCGA-DU-6403-01	Henry_Ford_Hospital	Brain_Lower_Grade_Glioma	oligoastrocytoma	G3	60	female	WT	non_codel	IDHwt	Unmethylated	Gain_chr_7_&_loss_chr_10	No_chr_19_20_gain	Mutant	TERT	0_400
TCGA-DU-6404-01	Henry_Ford_Hospital	Brain_Lower_Grade_Glioma	oligodendroglioma	G3	24	female	WT	non_codel	IDHwt	Unmethylated	No_combined_CNA	No_chr_19_20_gain	WT		400+
TCGA-DU-6405-01	Henry_Ford_Hospital	Brain_Lower_Grade_Glioma	astrocytoma	G3	51	female	WT	non_codel	IDHwt	Methylated	Gain_chr_7_&_loss_chr_10	No_chr_19_20_gain	Mutant	TERT	400+
TCGA-DU-6406-01	Henry_Ford_Hospital	Brain_Lower_Grade_Glioma	oligoastrocytoma	G3	59	female	WT	non_codel	IDHwt	Unmethylated	Gain_chr_7_&_loss_chr_10	Gain_chr_19_20	NA	NA	400+
TCGA-DU-6407-01	Henry_Ford_Hospital	Brain_Lower_Grade_Glioma	oligodendroglioma	G2	35	female	Mutant	non_codel	IDHmut_non_codel	Methylated	No_combined_CNA	No_chr_19_20_gain	WT	ATRX	400+
TCGA-DU-6408-01	Henry_Ford_Hospital	Brain_Lower_Grade_Glioma	oligodendroglioma	G3	23	female	Mutant	non_codel	IDHmut_non_codel	Methylated	No_combined_CNA	No_chr_19_20_gain	WT	ATRX	400+
TCGA-DU-7006-01	Henry_Ford_Hospital	Brain_Lower_Grade_Glioma	astrocytoma	G3	60	female	WT	non_codel	IDHwt	Methylated	Gain_chr_7_&_loss_chr_10	No_chr_19_20_gain	Mutant	TERT	0_400
TCGA-DU-7007-01	Henry_Ford_Hospital	Brain_Lower_Grade_Glioma	astrocytoma	G2	33	male	Mutant	non_codel	IDHmut_non_codel	Methylated	No_combined_CNA	No_chr_19_20_gain	WT	ATRX	400+
TCGA-DU-7010-01	Henry_Ford_Hospital	Brain_Lower_Grade_Glioma	astrocytoma	G3	58	female	Mutant	non_codel	IDHmut_non_codel	Methylated	No_combined_CNA	No_chr_19_20_gain	WT		400+
TCGA-DU-7012-01	Henry_Ford_Hospital	Brain_Lower_Grade_Glioma	astrocytoma	G3	74	female	WT	non_codel	IDHwt	Methylated	Gain_chr_7_&_loss_chr_10	No_chr_19_20_gain	Mutant	TERT	0_400
TCGA-DU-7013-01	Henry_Ford_Hospital	Brain_Lower_Grade_Glioma	astrocytoma	G3	59	male	WT	non_codel	IDHwt	Unmethylated	Gain_chr_7_&_loss_chr_10	No_chr_19_20_gain	Mutant	TERT	0_400
TCGA-DU-7014-01	Henry_Ford_Hospital	Brain_Lower_Grade_Glioma	oligodendroglioma	G2	59	male	NA	non_codel	NA	Methylated	No_combined_CNA	No_chr_19_20_gain	NA	NA	400+
TCGA-DU-7018-01	Henry_Ford_Hospital	Brain_Lower_Grade_Glioma	oligodendroglioma	G3	57	female	Mutant	codel	IDHmut_codel	Methylated	No_combined_CNA	No_chr_19_20_gain	Mutant	TERT	400+
TCGA-DU-7290-01	Henry_Ford_Hospital	Brain_Lower_Grade_Glioma	astrocytoma	G3	45	female	WT	non_codel	IDHwt	Unmethylated	No_combined_CNA	No_chr_19_20_gain	Mutant	TERT	0_400
TCGA-DU-7292-01	Henry_Ford_Hospital	Brain_Lower_Grade_Glioma	astrocytoma	G3	69	male	WT	non_codel	IDHwt	Methylated	No_combined_CNA	No_chr_19_20_gain	WT		0_400
TCGA-DU-7298-01	Henry_Ford_Hospital	Brain_Lower_Grade_Glioma	astrocytoma	G3	38	female	Mutant	non_codel	IDHmut_non_codel	Methylated	No_combined_CNA	No_chr_19_20_gain	WT	ATRX	400+
TCGA-DU-7299-01	Henry_Ford_Hospital	Brain_Lower_Grade_Glioma	astrocytoma	G3	33	male	Mutant	non_codel	IDHmut_non_codel	Methylated	No_combined_CNA	No_chr_19_20_gain	WT		400+
TCGA-DU-7300-01	Henry_Ford_Hospital	Brain_Lower_Grade_Glioma	oligodendroglioma	G3	53	female	Mutant	codel	IDHmut_codel	Methylated	No_combined_CNA	No_chr_19_20_gain	Mutant	TERT	400+
TCGA-DU-7301-01	Henry_Ford_Hospital	Brain_Lower_Grade_Glioma	oligodendroglioma	G2	53	male	Mutant	non_codel	IDHmut_non_codel	Methylated	No_combined_CNA	No_chr_19_20_gain	WT	ATRX	400+
TCGA-DU-7304-01	Henry_Ford_Hospital	Brain_Lower_Grade_Glioma	oligoastrocytoma	G3	43	male	Mutant	non_codel	IDHmut_non_codel	Methylated	No_combined_CNA	No_chr_19_20_gain	WT	ATRX	400+
TCGA-DU-8158-01	Henry_Ford_Hospital	Brain_Lower_Grade_Glioma	astrocytoma	G3	57	female	WT	non_codel	IDHwt	Unmethylated	No_combined_CNA	No_chr_19_20_gain	Mutant	TERT	0_400
TCGA-DU-8161-01	Henry_Ford_Hospital	Brain_Lower_Grade_Glioma	oligoastrocytoma	G3	63	female	WT	non_codel	IDHwt	Unmethylated	Gain_chr_7_&_loss_chr_10	No_chr_19_20_gain	Mutant	TERT	400+
TCGA-DU-A76L-01	Henry_Ford_Hospital	Brain_Lower_Grade_Glioma	oligodendroglioma	G3	54	male	WT	non_codel	IDHwt	Methylated	Gain_chr_7_&_loss_chr_10	No_chr_19_20_gain	NA	NA	400+
TCGA-DU-A76R-01	Henry_Ford_Hospital	Brain_Lower_Grade_Glioma	oligodendroglioma	G3	51	male	Mutant	codel	IDHmut_codel	Methylated	No_combined_CNA	No_chr_19_20_gain	NA	NA	400+
TCGA-E1-5302-01	Duke	Brain_Lower_Grade_Glioma	astrocytoma	G3	41	male	Mutant	non_codel	IDHmut_non_codel	Methylated	No_combined_CNA	No_chr_19_20_gain	WT	ATRX	400+
TCGA-E1-5303-01	Duke	Brain_Lower_Grade_Glioma	astrocytoma	G3	38	male	Mutant	non_codel	IDHmut_non_codel	Methylated	No_combined_CNA	No_chr_19_20_gain	WT	ATRX	400+
TCGA-E1-5304-01	Duke	Brain_Lower_Grade_Glioma	astrocytoma	G3	42	male	Mutant	non_codel	IDHmut_non_codel	Unmethylated	No_combined_CNA	No_chr_19_20_gain	WT	ATRX	400+
TCGA-E1-5305-01	Duke	Brain_Lower_Grade_Glioma	astrocytoma	G3	34	male	Mutant	non_codel	IDHmut_non_codel	Methylated	No_combined_CNA	No_chr_19_20_gain	WT	ATRX	400+
TCGA-E1-5307-01	Duke	Brain_Lower_Grade_Glioma	astrocytoma	G3	62	female	Mutant	non_codel	IDHmut_non_codel	Methylated	No_combined_CNA	No_chr_19_20_gain	WT	ATRX	400+
TCGA-E1-5311-01	Duke	Brain_Lower_Grade_Glioma	oligodendroglioma	G3	31	male	Mutant	codel	IDHmut_codel	Methylated	No_combined_CNA	No_chr_19_20_gain	Mutant	TERT	400+
TCGA-E1-5318-01	Duke	Brain_Lower_Grade_Glioma	oligodendroglioma	G2	42	female	Mutant	codel	IDHmut_codel	Methylated	No_combined_CNA	No_chr_19_20_gain	Mutant	TERT	400+
TCGA-E1-5319-01	Duke	Brain_Lower_Grade_Glioma	oligodendroglioma	G2	48	female	Mutant	codel	IDHmut_codel	Methylated	No_combined_CNA	No_chr_19_20_gain	Mutant	TERT	400+
TCGA-E1-5322-01	Duke	Brain_Lower_Grade_Glioma	oligoastrocytoma	G2	38	female	Mutant	non_codel	IDHmut_non_codel	Methylated	No_combined_CNA	No_chr_19_20_gain	Mutant	NA	400+
TCGA-HT-7607-01	Case_Western___St_Joes	Brain_Lower_Grade_Glioma	astrocytoma	G2	61	female	Mutant	codel	IDHmut_codel	Methylated	No_combined_CNA	No_chr_19_20_gain	Mutant	TERT	0_400
TCGA-HT-7616-01	Case_Western___St_Joes	Brain_Lower_Grade_Glioma	oligodendroglioma	G3	75	male	Mutant	codel	IDHmut_codel	Methylated	No_combined_CNA	No_chr_19_20_gain	Mutant	TERT	0_400
TCGA-HT-8013-01	Case_Western___St_Joes	Brain_Lower_Grade_Glioma	oligoastrocytoma	G2	37	female	Mutant	non_codel	IDHmut_non_codel	Methylated	No_combined_CNA	No_chr_19_20_gain	WT	ATRX	400+
TCGA-HT-A5RC-01	Case_Western___St_Joes	Brain_Lower_Grade_Glioma	astrocytoma	G3	70	female	WT	non_codel	IDHwt	Unmethylated	Gain_chr_7_&_loss_chr_10	Gain_chr_19_20	WT		0_400
TCGA-IK-7675-01	Christiana_Healthcare	Brain_Lower_Grade_Glioma	oligodendroglioma	G2	43	male	Mutant	non_codel	IDHmut_non_codel	Methylated	No_combined_CNA	No_chr_19_20_gain	WT	ATRX	400+
TCGA-06-0125-02	Henry_Ford_Hospital	Glioblastoma_multiforme	glioblastoma	G4	63	female	WT	non_codel	IDHwt	Methylated	No_combined_CNA	Gain_chr_19_20	Mutant	TERT	400+
TCGA-06-0152-02	Henry_Ford_Hospital	Glioblastoma_multiforme	glioblastoma	G4	68	male	WT	non_codel	IDHwt	NA	Gain_chr_7_&_loss_chr_10	No_chr_19_20_gain	NA	NA	0_400
TCGA-06-0171-02	Henry_Ford_Hospital	Glioblastoma_multiforme	glioblastoma	G4	65	male	WT	non_codel	IDHwt	NA	Gain_chr_7_&_loss_chr_10	No_chr_19_20_gain	Mutant	TERT	0_400
TCGA-06-0190-02	Henry_Ford_Hospital	Glioblastoma_multiforme	glioblastoma	G4	62	male	WT	non_codel	IDHwt	NA	Gain_chr_7_&_loss_chr_10	No_chr_19_20_gain	Mutant	TERT	0_400
TCGA-06-0210-02	Henry_Ford_Hospital	Glioblastoma_multiforme	glioblastoma	G4	72	female	WT	non_codel	IDHwt	NA	Gain_chr_7_&_loss_chr_10	No_chr_19_20_gain	Mutant	TERT	0_400
TCGA-06-0211-02	Henry_Ford_Hospital	Glioblastoma_multiforme	glioblastoma	G4	47	male	WT	non_codel	IDHwt	NA	Gain_chr_7_&_loss_chr_10	Gain_chr_19_20	Mutant	TERT	0_400
TCGA-06-0221-02	Henry_Ford_Hospital	Glioblastoma_multiforme	glioblastoma	G4	31	male	Mutant	non_codel	IDHmut_non_codel	NA	No_combined_CNA	No_chr_19_20_gain	WT	ATRX	400+
TCGA-06-1804-01	Henry_Ford_Hospital	Glioblastoma_multiforme	glioblastoma	G4	81	female	WT	non_codel	IDHwt	Methylated	Gain_chr_7_&_loss_chr_10	No_chr_19_20_gain	NA	NA	400+
TCGA-06-5408-01	Henry_Ford_Hospital	Glioblastoma_multiforme	glioblastoma	G4	54	female	WT	non_codel	IDHwt	Unmethylated	Gain_chr_7_&_loss_chr_10	No_chr_19_20_gain	NA	NA	0_400
TCGA-06-5410-01	Henry_Ford_Hospital	Glioblastoma_multiforme	glioblastoma	G4	72	female	WT	non_codel	IDHwt	Methylated	No_combined_CNA	No_chr_19_20_gain	NA	NA	0_400
TCGA-06-5411-01	Henry_Ford_Hospital	Glioblastoma_multiforme	glioblastoma	G4	51	male	WT	non_codel	IDHwt	Unmethylated	Gain_chr_7_&_loss_chr_10	No_chr_19_20_gain	Mutant	TERT	0_400
TCGA-06-5412-01	Henry_Ford_Hospital	Glioblastoma_multiforme	glioblastoma	G4	78	female	WT	non_codel	IDHwt	Methylated	No_combined_CNA	No_chr_19_20_gain	NA	NA	0_400
TCGA-06-5418-01	Henry_Ford_Hospital	Glioblastoma_multiforme	glioblastoma	G4	75	female	WT	non_codel	IDHwt	Unmethylated	Gain_chr_7_&_loss_chr_10	No_chr_19_20_gain	NA	NA	0_400
TCGA-06-5856-01	Henry_Ford_Hospital	Glioblastoma_multiforme	glioblastoma	G4	58	male	WT	non_codel	IDHwt	Unmethylated	Gain_chr_7_&_loss_chr_10	No_chr_19_20_gain	NA	NA	0_400
TCGA-12-5295-01	Duke	Glioblastoma_multiforme	glioblastoma	G4	60	female	WT	non_codel	IDHwt	Methylated	Gain_chr_7_&_loss_chr_10	No_chr_19_20_gain	NA	NA	400+
TCGA-12-5299-01	Duke	Glioblastoma_multiforme	glioblastoma	G4	56	female	WT	non_codel	IDHwt	Unmethylated	Gain_chr_7_&_loss_chr_10	No_chr_19_20_gain	NA	NA	0_400
TCGA-14-0736-02	Emory_University	Glioblastoma_multiforme	glioblastoma	G4	49	male	NA	non_codel	NA	Unmethylated	Gain_chr_7_&_loss_chr_10	No_chr_19_20_gain	NA	NA	400+
TCGA-14-0781-01	Emory_University	Glioblastoma_multiforme	glioblastoma	G4	49	male	WT	non_codel	IDHwt	Unmethylated	No_combined_CNA	No_chr_19_20_gain	NA	NA	0_400
TCGA-14-1034-02	Emory_University	Glioblastoma_multiforme	glioblastoma	G4	60	female	WT	non_codel	IDHwt	Methylated	Gain_chr_7_&_loss_chr_10	No_chr_19_20_gain	Mutant	TERT	400+
TCGA-14-1402-02	Emory_University	Glioblastoma_multiforme	glioblastoma	G4	58	female	NA	non_codel	NA	Methylated	No_combined_CNA	Gain_chr_19_20	Mutant	NA	400+
TCGA-15-1444-01	Mayo_Clinic___Rochester	Glioblastoma_multiforme	glioblastoma	G4	21	male	Mutant	non_codel	IDHmut_non_codel	Methylated	No_combined_CNA	No_chr_19_20_gain	WT	ATRX	400+
TCGA-19-0957-02	Case_Western	Glioblastoma_multiforme	glioblastoma	G4	48	female	WT	non_codel	IDHwt	Unmethylated	Gain_chr_7_&_loss_chr_10	No_chr_19_20_gain	NA	NA	400+
TCGA-19-1389-02	Case_Western	Glioblastoma_multiforme	glioblastoma	G4	51	male	WT	non_codel	IDHwt	Unmethylated	Gain_chr_7_&_loss_chr_10	No_chr_19_20_gain	Mutant	NA	0_400
TCGA-26-5135-01	University_of_Florida	Glioblastoma_multiforme	glioblastoma	G4	72	female	WT	non_codel	IDHwt	Methylated	Gain_chr_7_&_loss_chr_10	No_chr_19_20_gain	Mutant	TERT	0_400
TCGA-28-5204-01	Cedars_Sinai	Glioblastoma_multiforme	glioblastoma	G4	72	male	WT	non_codel	IDHwt	Unmethylated	Gain_chr_7_&_loss_chr_10	No_chr_19_20_gain	NA	NA	400+
TCGA-28-5207-01	Cedars_Sinai	Glioblastoma_multiforme	glioblastoma	G4	71	male	WT	non_codel	IDHwt	Unmethylated	Gain_chr_7_&_loss_chr_10	No_chr_19_20_gain	NA	NA	0_400
TCGA-28-5215-01	Cedars_Sinai	Glioblastoma_multiforme	glioblastoma	G4	62	female	WT	non_codel	IDHwt	Methylated	No_combined_CNA	No_chr_19_20_gain	NA	NA	0_400
TCGA-28-5218-01	Cedars_Sinai	Glioblastoma_multiforme	glioblastoma	G4	63	male	WT	non_codel	IDHwt	Unmethylated	No_combined_CNA	Gain_chr_19_20	NA	NA	0_400
TCGA-32-1980-01	St._Joseph's_Hospital_(AZ)	Glioblastoma_multiforme	glioblastoma	G4	72	male	WT	non_codel	IDHwt	Unmethylated	No_combined_CNA	No_chr_19_20_gain	NA	NA	0_400
TCGA-76-4925-01	Thomas_Jefferson_University	Glioblastoma_multiforme	glioblastoma	G4	76	male	WT	non_codel	IDHwt	Methylated	Gain_chr_7_&_loss_chr_10	No_chr_19_20_gain	NA	NA	0_400
TCGA-76-4926-01	Thomas_Jefferson_University	Glioblastoma_multiforme	glioblastoma	G4	68	male	WT	non_codel	IDHwt	Unmethylated	Gain_chr_7_&_loss_chr_10	No_chr_19_20_gain	NA	NA	0_400
TCGA-76-4927-01	Thomas_Jefferson_University	Glioblastoma_multiforme	glioblastoma	G4	58	male	WT	NA	NA	Unmethylated	NA	NA	NA	NA	400+
TCGA-76-4928-01	Thomas_Jefferson_University	Glioblastoma_multiforme	glioblastoma	G4	85	female	WT	non_codel	IDHwt	Methylated	Gain_chr_7_&_loss_chr_10	No_chr_19_20_gain	NA	NA	0_400
TCGA-76-4929-01	Thomas_Jefferson_University	Glioblastoma_multiforme	glioblastoma	G4	76	female	WT	non_codel	IDHwt	Methylated	Gain_chr_7_&_loss_chr_10	No_chr_19_20_gain	NA	NA	0_400
TCGA-76-4931-01	Thomas_Jefferson_University	Glioblastoma_multiforme	glioblastoma	G4	70	female	WT	non_codel	IDHwt	Unmethylated	Gain_chr_7_&_loss_chr_10	No_chr_19_20_gain	NA	NA	0_400
TCGA-76-4932-01	Thomas_Jefferson_University	Glioblastoma_multiforme	glioblastoma	G4	50	female	WT	NA	NA	Methylated	NA	NA	NA	NA	400+

Codes:

MCFS part (R code):

```
##########################################################################################

#read the data from the internet

#this is TCGA data for brain tumor patients

brain_cancer_data <-
readRDS(file("http://zbo.ipipan.waw.pl/files/supplementary/Scientific_Reports_2018/G2_G3_G4_v1.rds", "rb"))

dim(brain_cancer_data)

brain_cancer_data

table(cut(brain_cancer_data$death_days_to, c(0,400,1000,5000)))

table(brain_cancer_data$decision)


#remove patient ID and continous survival time

brain_cancer_data <- brain_cancer_data[,!names(brain_cancer_data) %in% c("ID","death_days_to")]


#install rmcfs from CRAN repository

install.packages('rmcfs')

options(java.parameters = "-Xmx16g")

library(rmcfs)


#run mcfs on data

#be patient it can take couple of hours - it depends on your machine

#set up threadsNumber it maximum value should equal to your cores number

brain_cancer_result <- mcfs(decision~., brain_cancer_data, projections = 50000, projectionSize = 500,

                    cutoffPermutations = 20, threadsNumber = 12)


#review the result of the analysis

brain_cancer_result
```

```
brain_cancer_result$cutoff

head(brain_cancer_result$RI, brain_cancer_result$cutoff_value)


plot(brain_cancer_result, type = "features")

plot(brain_cancer_result, type = "cv", cv_measure = "wacc")

plot(brain_cancer_result, type = "heatmap", heatmap_norm = 'norm', heatmap_fun = 'median')



#load top 5k highly estimated features together with clinical data

brain_cancer_data_top_5k <-
readRDS(file("http://zbo.ipipan.waw.pl/files/supplementary/Scientific_Reports_2018/G2G3G4_v1_data.PatientData.5k.
rds", "rb"))

dim(brain_cancer_data_top_5k)

table(brain_cancer_data_top_5k$decision)


#run mcfs on this data

brain_cancer_result_5k <- mcfs(class~., brain_cancer_data_top_5k, projections = 1000, projectionSize = 50,
cutoffPermutations = 20, threadsNumber = 12)


plot(brain_cancer_result_5k, type = "features")

plot(brain_cancer_result_5k, type = "cv", cv_measure = "wacc")

plot(brain_cancer_result_5k, type = "heatmap", heatmap_norm = 'norm', heatmap_fun = 'median')

###########################################################################################
```

Mutual Information code:

https://www.dropbox.com/sh/7c6qzqb1pf8kpfg/AAAtZCkDhrWJ9T9Ffvu4T_fQa?dl=0

References

1. McGill, W. J. Multivariate information transmission. *Psychometrika* **19,** 97–116 (1954).

2. Moore, J. H. & M., W. S. *Epistasis*. **1253,** (Springer New York, 2015).

3. Mielniczuk, J. & Rdzanowski, M. Use of information measures and their approximations to detect predictive gene-gene interaction. *Entropy* **19,** (2017).

4. Zhang, Y. I-TASSER server for protein 3D structure prediction. *BMC Bioinformatics* **9,** 40 (2008).

5. Jones, D. T. & Swindells, M. B. Getting the most from PSI–BLAST. (2002).

6. Tuszynska, I., Magnus, M., Jonak, K., Dawson, W. & Bujnicki, J. M. NPDock: a web server for protein–nucleic acid docking. *Nucleic Acids Res.* **43,** W425–W430 (2015).

7. Lietz, M., Hohl, M. & Thiel, G. RE-1 silencing transcription factor (REST) regulates human synaptophysin gene transcription through an intronic sequence-specific DNA-binding site. *FEBS J.* **270,** 2–9 (2003).

8. Dunbrack, R. L. & Cohen, F. E. Bayesian statistical analysis of protein side-chain rotamer preferences. *Protein Sci.* **6,** 1661–1681 (1997).

9. Krieger, E. & Vriend, G. New ways to boost molecular dynamics simulations. *J. Comput. Chem.* **36,** 996–1007 (2015).

10. Li, J. *et al.* Identification of high-quality cancer prognostic markers and metastasis network modules. *Nat. Commun.* **1,** 34 (2010).

11. Roy, A., Kucukural, A. & Zhang, Y. I-TASSER: a unified platform for automated protein structure and function prediction. *Nat. Protoc.* **5,** 725–738 (2010).
